# A pro-angiogenic and hypoxic zebrafish model as a novel platform for anti-angiogenic drug testing

**DOI:** 10.1242/bio.061863

**Published:** 2025-08-11

**Authors:** Vinoth S., Kirankumar Santhakumar

**Affiliations:** Zebrafish Genetics Laboratory, Department of Genetic Engineering, SRM Institute of Science and Technology, Kattankulathur 603 203, Tamil Nadu, India

**Keywords:** Angiogenesis, Hypoxia, Drug testing, Zebrafish model, Kinase inhibitor

## Abstract

Zebrafish is a valuable model for antiangiogenic drug testing. We hypothesized that the efficacy of antiangiogenic compounds might vary in hypoxic tissue environments compared to normal tissue. To explore this, we established a chemically induced zebrafish model using DMOG, which inhibits prolyl hydroxylases, and a genetic model by knocking out *vhl* gene via CRISPR/Cas9 to activate hypoxia signaling. In wild-type larvae, the antiangiogenic drug sorafenib inhibited blood vessel growth. However, in the DMOG model and *vhl^−/−^* model, no inhibition occurred in sub-intestinal vessel (SIV) upon sorafenib treatment. Also, gene expression analysis showed that the DMOG induced hypoxia had 20-fold increase in *phd3* expression, a marker for hypoxia signaling activation, which rose to 65-fold and 280-fold with sorafenib treatment at the concentration 0.1 μM and 0.2 μM, respectively. In the *vhl^−/−^* model *phd3* expression was found to be increased to 220-fold and reaching up to 400-fold with sorafenib treatment. This increased activation of hypoxia signaling elevated the proangiogenic factors like *vegfaa*, *vegfab* and *vegfd*, which might have protected the SIV region from sorafenib treatment in hypoxic models. This confirms that the hypoxia zebrafish models gained resistance against chemotherapeutic drugs by increasing the cellular hypoxia levels. Thus, our zebrafish model for hypoxia provides evidence that the efficacy of chemotherapy for cancer significantly depends on hypoxic microenvironment.

## Teaser

**Summary:** We present a drug resistant pro-angiogenic zebrafish model to test potential anti-angiogenic drugs for anti-cancer therapeutics.

## INTRODUCTION

Angiogenesis, the formation of new blood vessels, is a critical aspect of cancer progression, supporting the growth of tumor cells by supplying nutrients and signaling molecules (Lugano et al., 2020). Identifying effective antiangiogenic drugs is crucial for cancer treatment. Zebrafish is a widely used *in vivo* model to analyses the pro- and antiangiogenic effect of any experimental compound/drug (Santoro, 2014). In general, the zebrafish embryos are treated at 1 or 2 dpf and the antiangiogenic property of the drug is identified by looking at the intersegmental vessel at 2 dpf and sub intestinal vessels at 3 dpf larvae, respectively, after 24 or 48 h of treatment (Chen et al., 2015). But with this procedure the efficacy of the drug is tested in normal zebrafish larvae where the levels of hypoxia signaling are low, while in most of the solid tumors, intratumoral hypoxia is a common phenomenon and hypoxia-inducible factor (HIF) signaling pathway is the major regulator of angiogenesis (Gossage et al., 2014).

In normal conditions, the experimental compound may show promising results in zebrafish models, suggesting potential therapeutic benefits. However, the transition to hypoxic conditions in human tumor patients introduces a complex physiological microenvironment. This altered condition can significantly impact the compound's ability to exert its intended effects. We hypothesized that although the compound may demonstrate efficacy under normal/normoxic conditions in zebrafish, its effectiveness could vary considerably when administered to patients experiencing hypoxia.

Hypoxia is characterized by a reduction in normal oxygen levels in tissues. During hypoxia, cells adapt to survive in low oxygen environments, and the oxygen supply to hypoxic sites is enhanced through the formation of new blood vessels (Tirpe et al., 2019). These adaptive responses are mediated by a transcription factor known as HIF. Under normal oxygen conditions, HIF-α monomer is hydroxylated by an enzyme called prolyl hydroxylase (PHD) and undergoes ubiquitin-mediated degradation via the von Hippel Lindau (VHL) complex. However, in hypoxic conditions where oxygen levels are low, HIF-α is not hydroxylated by PHD. As a result, it does not bind to the VHL complex, leading to the stabilization of HIF-α. Stabilized HIF-α then forms a heterodimer with HIF-β, which initiates early transcriptional responses by binding to hypoxia-responsive elements (HREs) (Gossage et al., 2014). This activation leads to the expression of various genes involved in adaptive responses to hypoxia. These genes include *VEGF*, which promotes angiogenesis; *EPO*, which stimulates red blood cell synthesis; *GLUT1*, which is involved in glucose metabolism; and several other target genes specific to both normal and cancer cells (such as clear cell renal cell carcinoma, hepatocellular carcinoma). This orchestrated response helps cells to cope with and adapt to low oxygen conditions, thereby promoting survival and function in hypoxic environments (Tirpe et al., 2019).

Therefore, our hypothesis posits that the compound's efficacy, which appeared promising under normoxic conditions in zebrafish, may be diminished or altered when confronted with the challenges posed by hypoxic conditions in human patients. In our study, we aimed to evaluate the efficacy of an antiangiogenic drug under activated hypoxic conditions in zebrafish, comparing the outcomes to normal conditions. There are three methods to activate hypoxia signaling in zebrafish: (1) by exposing zebrafish larvae to low oxygen environments, triggering activation of the hypoxia pathway due to reduced oxygen levels. (2) By inhibiting the function of PHD, thereby preventing hydroxylation of Hifα even under normal oxygen levels. This inhibition prevents ubiquitin-mediated degradation of HIF-α mediated by the VHL complex. And (3) by directly inactivating the function of VHL, leading to stabilization of HIF-α and preventing its degradation.

The first method is a true hypoxia state because the lack of oxygen leads to HIF-α stabilization and activation of the hypoxia signaling pathway in zebrafish larvae. The other two are pseudo-hypoxia conditions because the hypoxia signaling is activated even in the presence of oxygen. Out of these three methods, we chose pseudo-hypoxia conditions for the activation of hypoxia signaling in zebrafish larvae to test our hypothesis. (I) A chemical compound called dimethyloxalylglycine (DMOG), which is well-known for its ability to inhibit PHD and activate the hypoxia signaling pathway in zebrafish larvae (van Rooijen et al., 2009; Santhakumar et al., 2012). (II) We have created a *vhl* knockout mutant zebrafish using CRISPR-Cas9 technique, which exhibits robust activation of HIF signaling. For antiangiogenic testing, we selected sorafenib, a multikinase inhibitor known for its antiangiogenic and antiproliferative effects (Adnane et al., 2006). In this study, we exposed DMOG-induced pseudo-hypoxic larvae, *vhl^−/−^* mutant and wild-type larvae to sorafenib to test our hypothesis. Sorafenib targets multiple intracellular serine/threonine kinases within the Ras/mitogen-activated protein kinase (MAPK) signaling pathway (Adnane et al., 2006; Wilhelm et al., 2006). In particular, it inhibits the wild-type B-Raf, the mutant B-Raf, and Raf-1 (C-Raf), among other isoforms of Raf serine/threonine kinases. Furthermore, sorafenib inhibits a number of cell surface tyrosine kinase receptors, including platelet-derived growth factor receptor-β (PDGFR-β), vascular endothelial growth factor receptors (VEGFR-1, VEGFR-2, and VEGFR-3), KIT, FMS-like tyrosine kinase 3 (FLT-3), RET, and RET/PTC (Keating, 2017; Liu et al., 2006). Hence in this study, we aimed to assess how sorafenib functions under conditions of activated hypoxia signaling pathway compared to normal conditions, providing insights into its potential effectiveness in treating cancer conditions where hypoxia is a significant factor in tumor angiogenesis.

## RESULTS

### Accelerated development of sub-intestinal vessel (SIV) in DMOG treated zebrafish larvae

DMOG has the potential to inhibit PHDs and to stabilize the HIF-α under *in vitro* and *in vivo* conditions (Sen and Sen, 2016). Previous reports suggest that zebrafish larvae (from 2 dpf) could survive up to 100 μM treatment of DMOG, which would result in activation of the hypoxia signaling pathway (van Rooijen et al., 2009). We initiated treatment at 2 dpf stage of *Tg(fli1a*:*EGFP)^y1^* in zebrafish larvae and assessed changes in the SIV pattern at 3 dpf (Fig. S2). According to previous literature (Chen et al., 2015), we anticipated an angiogenic sprouting phenotype or extension of the SIV region due to the proangiogenic effects of hypoxia-inducing drugs.

Fig. S1 illustrates the typical phenotype of the SIV in untreated zebrafish larvae, as well as the effects of DMOG treatment. Normally, at 3 dpf, control larvae exhibit a specific SIV pattern, which is typically seen developing into a ‘V’ shape by 4 dpf, as shown in Fig. S1. However, larvae treated with DMOG showed an accelerated development of this V-shaped SIV pattern, which was noticeable as early as 3 dpf (Fig. S1). Additionally, we observed mild blood flow movement over the liver, which is unusual for this developmental stage in DMOG treated group (Movie 1).

### Observation of liver angiogenesis in zebrafish larvae through *O*-dianisidine staining

To investigate blood vessel development in the liver, we adopted and developed a method based on *O*-dianisidine staining. *O*-dianisidine is a peroxidase activity indicator that stains the hemoglobin in distinct orange-ish-red color (Brien, 1961)_._ The zebrafish larvae were stained with *O*-dianisidine to detect the presence of hemoglobin cells, which helped assess hematopoiesis at the 2 and 3 dpf stages (Detrich et al., 1995; van Rooijen et al., 2009). During the staining procedure, the larvae were fixed with 4% paraformaldehyde (PFA). Once the larvae were fixed, the blood cells started to accumulate in the heart and nearby blood vessels. During the staining procedure, we found an accumulation of blood cells just posterior to the heart and liver regions in the 4 dpf zebrafish larvae when observed in a simple compound microscope (Fig. S2). After imaging, we observed a clear visualization of the blood vessel pattern in the liver region due to blood cell accumulation (Fig. S2).

When comparing control and DMOG-treated larvae at 4.5 dpf, we observed two distinct outcomes: (1) the 30% of larvae (three out of ten) exhibited excessive vessel growth, as shown in Fig. S2I-c1, with an extended SIV region but no excessive liver angiogenesis. (2) 70% of larvae displayed excessive angiogenesis in the liver, as depicted in Fig. S2II, but no extension of the SIV region. This suggests a clear relationship of excessive liver angiogenesis correlating with the absence of SIV extension, and vice versa. The results indicate that pro-angiogenic compound treatment leads to either extensive SIV development or excessive liver angiogenesis, but not both simultaneously.

**Fig. 1.**
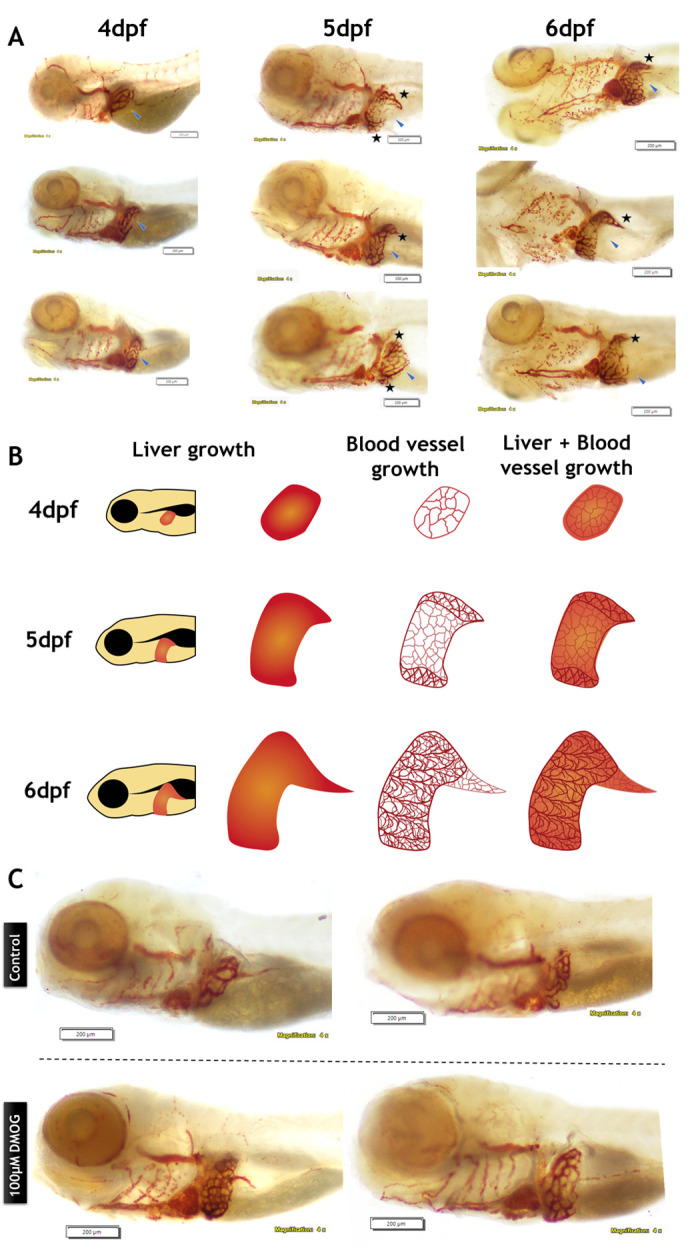
**Observation of liver angiogenesis in larval stages of zebrafish.** (A) The liver blood-vessel development pattern in zebrafish larvae at 4 dpf, 5 dpf, and 6 dpf. (B) Diagrammatic representation of liver growth and blood vessel development pattern in zebrafish larvae. (C) Observation of liver angiogenesis in control and DMOG-treated zebrafish larvae stained with *O-*dianisidine.

The observed excessive angiogenesis in the liver of DMOG-treated larvae appeared to resemble an early angiogenic pattern. Comparing this DMOG-induced liver angiogenic phenotype with the liver angiogenic patterns of wild-type larvae at subsequent developmental stages could help determine whether it represents neo-vasculature or an early growth of blood vessels over the liver region. Additionally, to enable a direct comparison of liver angiogenic patterns in the DMOG group, it is important to note that no previous studies have reported liver angiogenic patterns in zebrafish larvae after 4 dpf. So, by implementing this idea of *O*-dianisidine staining, we observed blood vessel development patterns in the liver of 4 to 6 dpf zebrafish larvae (Fig. 1A). Our analysis of these patterns revealed that blood vessels initially cover the posterior region of the liver at 4 dpf larval stage (Fig. 1A). At the 5 dpf stage, we observed that the blood vessel network extended from the existing vessels. At the 5 dpf stage larvae, we observed inward, front-side folding of blood vessels undergoing migration/extension (Fig. 1A). This inward front folding at 5 dpf, and further blood vessel extension at 6 dpf, clearly indicates that initial vascularization took place at the posterior side of the liver. Then the blood vessel network extended to the anterior side of the liver in 5 dpf zebrafish larva and covered the whole of the liver region by 6 dpf (Fig. 1A).

A diagram depicting the liver angiogenic pattern from 4 to 6 dpf in zebrafish larvae has been included to provide further clarity (Fig. 1B). Finally, we treated zebrafish larvae with 100 μM DMOG from 2 dpf and observed the liver blood-vessel pattern changes at 4 dpf, which showed increased blood vessel growth in the entire liver (Fig. 1C).

### Effect of an anti-angiogenic drug on the DMOG-induced pseudo-hypoxia zebrafish model

The chemical proangiogenic model exhibited higher angiogenesis in the liver after 2.5 days of DMOG treatment. Hence, this chemical pseudo-hypoxia model was ready to test our hypothesis that a compound/drug might show promise under normal conditions in zebrafish, but its efficacy could differ significantly when treated under hypoxia.

**Fig. 2.**
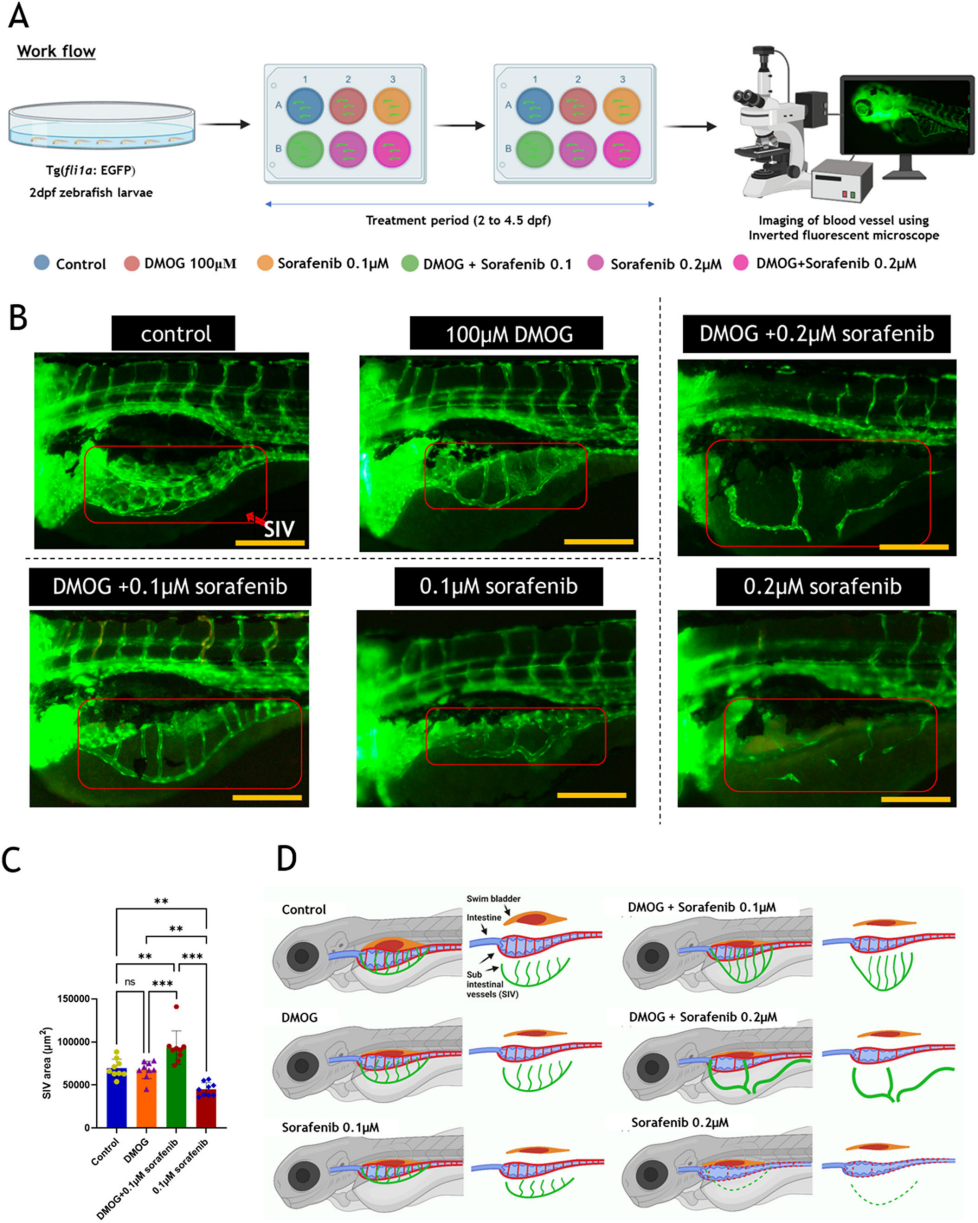
**Effect of sorafenib on DMOG-induced pseudo-hypoxia zebrafish larvae.** (A) Diagrammatic representation of steps followed in sorafenib treatment on DMOG-induced proangiogenic pseudo-hypoxia model. (B) Images showing changes in SIV region of zebrafish larvae *Tg(fli1a*:*EGFP)* at 4.5 dpf. Scale bars: 200 uM. (C) Measurement of SIV area (*n*=9). One-way ANOVA, Tukey's post-hoc test, Error bar: Mean±s.d.; **P≤0.01, ***P≤0.001; ns, non-significant. (D) Diagrammatic representation of changes observed in SIV region of zebrafish larvae at 4.5 dpf. Created in BioRender by Chakraborty, P., 2025. https://BioRender.com/kziqy4f. This figure was sublicensed under CC-BY 4.0 terms.

We treated both hypoxic and normoxic zebrafish larvae with sorafenib, a tyrosine kinase inhibitor recognized for its anti-angiogenic properties (Fig. 2A). In this experiment, initially, four groups were analyzed: control group (untreated), sorafenib-alone group (0.1 μM sorafenib), sorafenib plus DMOG group (0.1 μM sorafenib plus 100 μM DMOG treated at the same time), and DMOG-alone group. In the control group, we observed the normal extension and dense network of blood vessels in the SIV region (Fig. 2B). In the DMOG group, a clear SIV vessel was observed with a slightly smaller extension and much reduced angiogenic network than the control (Fig. 2B). When treated with 0.1 μM sorafenib alone, the existing structure of the SIV vessel was unaffected, but its extension was observed to be blocked and located centrally in the larval body as depicted in Fig. 2B. In the 0.1 μM sorafenib+DMOG-treated group, as shown in Fig. 2B, the SIV extension was longer than in the control, DMOG alone, or 0.1 μM sorafenib groups. When 0.1 μM sorafenib was administered in the zebrafish larvae, it limited the extension seen in the SIV region (Fig. 2C). The sorafenib plus DMOG co-treatment group larvae exhibited the extended SIV region phenotype in 100% of larvae and not the liver angiogenic phenotype as we observed in the DMOG treatment. Upon sorafenib treatment under the DMOG-induced pseudo-hypoxia condition, the larvae overcame limitation to SIV extension phenotype and the absence of liver angiogenesis in all the treated larvae.

Subsequently, we aimed to increase the concentration to 0.2 μM sorafenib and to observe its effects in both control and DMOG-induced hypoxic models. When sorafenib was administered alone at 0.2 μM, it completely inhibited the formation of the SIV region, with only a few endothelial cells observed, as depicted in Fig. 2B. In the 0.2 μM sorafenib+DMOG treated group, inhibition in the SIV region was also observed but not to the same extent. Fig. 2B shows an incompletely formed, dilated, and highly extended SIV in the 0.2 μM sorafenib+DMOG treated group. From these two different concentrations of sorafenib treatment, we conclude that when sorafenib is administered under pseudo-hypoxia background the effect of sorafenib is suppressed. At lower concentrations of 0.1 μM sorafenib, the pseudo-hypoxia condition successfully countered its effects. However, at the increased concentration of 0.2 μM sorafenib, the pseudo-hypoxic zebrafish larvae did not completely counteract the drug's effects. Nonetheless, as observed in Fig. 2B,C and D the pseudo-hypoxia condition clearly combats the effects of sorafenib.

For clarity on SIV blood vessel patterns across all groups, a diagram is provided in Fig. 2D. As previously noted, the control group exhibited a dense network of blood vessels in the SIV region. In Fig. 2B, the swim bladder inflation is evident, which displaces the intestine slightly downward from the body's midpoint. Consequently, two layers of SIVs overlap the intestine: one layer, marked in red, directly covers the intestine, while another layer in green, overlaps the previous layer of blood vessels (Fig. 2D). This overlapping results in an exceptionally dense network over the SIV region (Fig. 2D). In the DMOG group, zebrafish larvae failed to inflate the swim bladder, thus positioning the intestine centrally within the body. Here, the SIV in green extends slightly downward, without overlapping layers of blood vessels. This configuration allows for a clear observation of the SIV in the DMOG group (Fig. 2D). Similarly, in the 0.1 μM sorafenib group, swim bladder inflation is absent. However, due to the drug's antiangiogenic properties, the SIV is precisely restricted to the area where the intestine is located (Fig. 2D).

In contrast, the co-treatment group with 0.1 μM sorafenib plus DMOG exhibits significantly extended vessels compared to either the DMOG or 0.1 μM sorafenib alone treated groups (Fig. 2C). This suggests that pseudo-hypoxia may counteract the effects of sorafenib. The heightened extension of vessels in the 0.1 μM sorafenib plus DMOG group compared to the DMOG-alone treated group indicates a potential increase in cellular hypoxia levels. Furthermore, the 0.2 μM sorafenib group demonstrated complete inhibition of both layers of SIV (Fig. 2C), while co-treatment with DMOG did not completely inhibit SIV formation (Fig. 2C). Instead, the SIV region exhibited incomplete and dilated blood vessels, as depicted in Fig. 2C and D. From this experiment, we infer that the DMOG-induced pseudo-hypoxia leads to partial drug resistance against both 0.1 μM and 0.2 μM sorafenib. Therefore, we proceeded to investigate the levels of hypoxia signaling activation, endothelial growth factor expression, and their receptor levels to gain a deeper understanding of their potential molecular interactions.

### DMOG-exposed larvae exhibit resistance against sorafenib treatment through HIF activation

**Fig. 3.**
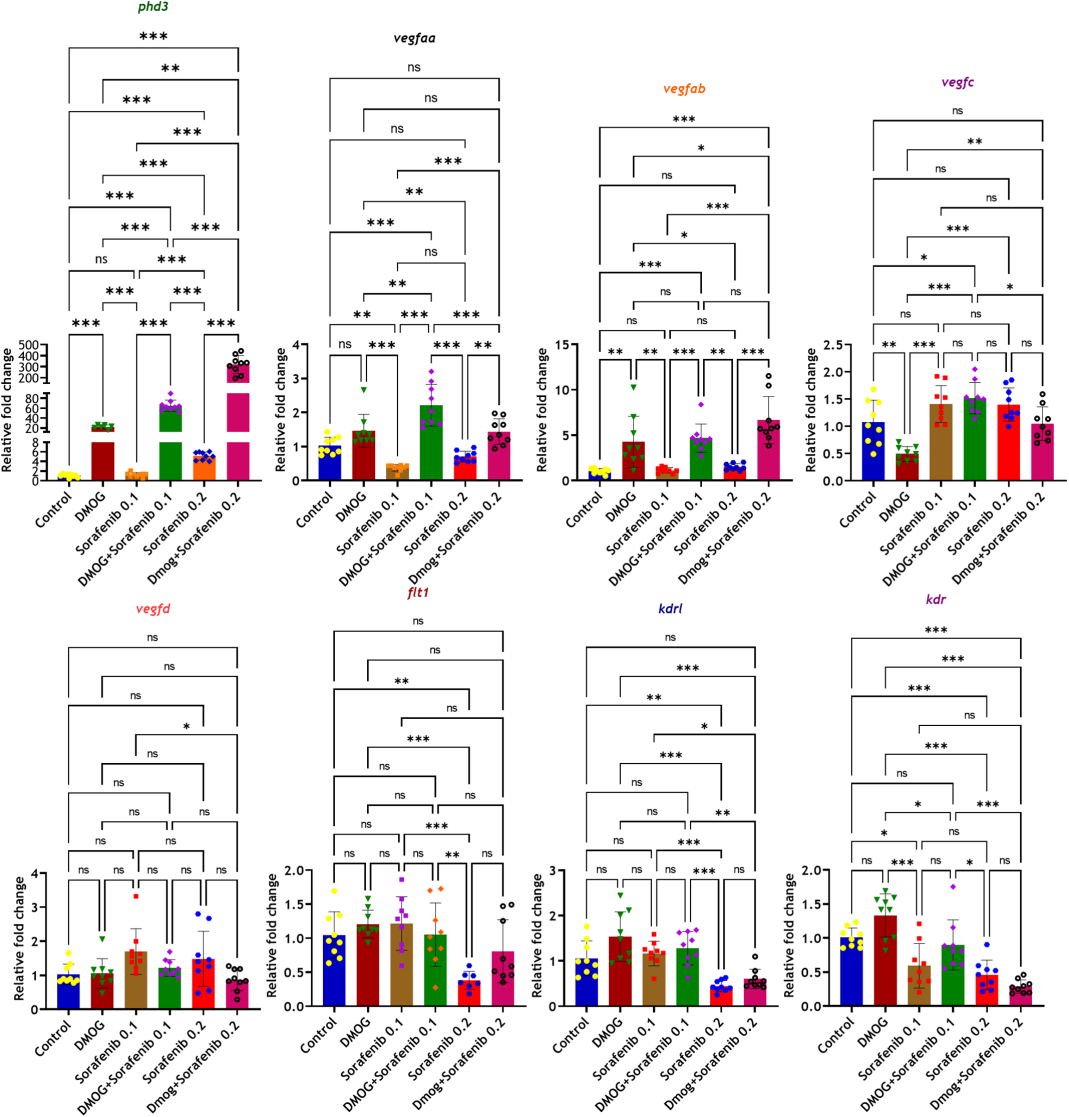
**Comparative gene expression analysis by RT-qPCR.** 50 larvae per each well, biological triplicate for each group, for each biological triplicate three technical triplicates were kept in RT PCR. *n*=biological triplicate*technical triplicate=3*3=9 values for each group. One-way ANOVA, Tukey's post-hoc test, Error bar: Mean±s.d.; *P≤0.05, **P≤0.01, ***P≤0.001; ns, non-significant.

Initially, our objective was to assess hypoxia levels across all experimental groups by examining the expression of the gene *phd3*. It has been established that *phd3* expression increases with stabilization of HIF-α, thus serving as an indicator of hypoxia signaling levels (van Rooijen et al., 2009). Compared to the control group, the DMOG group exhibited an average 20-fold increase in *phd3* expression, confirming that DMOG effectively enhances hypoxia levels in zebrafish larvae (Fig. 3). Conversely, the 0.1 μM sorafenib treated group did not show a significant difference in *phd3* expression compared to the control (Fig. 3). However, the co-treatment group with 0.1 μM sorafenib plus DMOG demonstrated a remarkable 65-fold increase in *phd3* expression relative to the control (Fig. 3). The enhanced activation of the HIF pathway, as indicated by *phd3* expression, might result in higher VEGF signaling in the treated larvae. This substantial increase in HIF and VEGF activation might contribute to overcoming the effects of sorafenib in the larvae experiencing pseudo-hypoxia. Consequently, the observed highly extended SIV in the 0.1 μM sorafenib plus DMOG co-treatment group correlates with the increased hypoxia signaling levels as indicated by *phd3* expression.

In the 0.2 μM sorafenib treated group, there was a notable 5-fold increase in *phd3* expression compared to the control, attributed to the complete inhibition of blood vessel formation in the SIV region (Fig. 3). In contrast, the co-treatment group of 0.2 μM sorafenib plus DMOG displayed a significant 300-fold increase in *phd3* expression relative to the control (Fig. 3). This substantial increase underscores the pronounced hypoxia signaling activation induced by DMOG, which is likely responsible for the incomplete and dilated formation of SIV observed in Fig. 2B and C. The failure of DMOG treatment to completely overcome the inhibition of angiogenesis in the SIV region during 0.2 μM sorafenib co-treatment can be attributed to the persistent and intensified hypoxia levels, which reach as high as 300-fold. These findings highlight that in the co-treatment scenarios, the DMOG induced hypoxic signaling is able to overcome the antiangiogenic effect of 0.1 μM sorafenib treatment. But the DMOG mediated HIF activation is not sufficient to counteract the effect of 0.2 μM sorafenib, resulting in incomplete inhibition of angiogenesis in the SIV region (Fig. 2B).

The gene expression analysis indicated that DMOG plus sorafenib treated group showed a notable increase in the expression levels of VEGFs such as *vegfaa* (DMOG+0.1 μM sorafenib) and *vegfab* (DMOG+0.1 μM sorafenib, DMOG+0.2 μM sorafenib) compared to the control group (Fig. 3). As per the cycle threshold (CT) value in RT-qPCR analysis, we observed that *vegfaa* exhibited the highest expression across all types of *vegf* in zebrafish, while *vegfab* was specifically observed to be expressed under hypoxic conditions. The *vegfc* was found to be the ranked as the second most expressed VEGF isoform after *vegfaa*. In the DMOG group, there was a significant downregulation of *vegfc* (Fig. 3). The *vegfd* showed unchanged expression across all treatment conditions (Fig. 3). Notably, DMOG treatment did not show significant differences in the expression levels of VEGF receptors such as *kdrl*, *kdr*, and *flt1* (Fig. 3). Conversely, treatment with 0.2 μM sorafenib resulted in a significant downregulation of these receptors, whereas co-treatment with DMOG did not significantly restore their expression levels compared to the control.

### Generation of genetic pseudo-hypoxia model to test sorafenib treatment resistance phenotype

#### Screening of deleterious and structural destabilizing nsSNPs in *VHL*

Using computational tools and structural biology approaches we predicted the most impactful missense variant positions in pVHL protein sequence. In total 325 nsSNPs obtained from the Ensembl database were subjected to analysis using eight prediction tools: SIFT, PolyPhen-2, PANTHER, PROVEAN, PMUT, SNP&GO, Align-GVGD, and MUTPRED. Among these nsSNPs, 116 were identified as common deleterious or disease-associated variants across the prediction tools (Fig. S3). Subsequently, all these 116 deleterious variants underwent protein stability analysis through tools such as mCSM, MUpro, iMUTANT, iSTABLE, SDM, CUPSAT. Among these variants 55 nsSNPs commonly were identified as capable of destabilizing the structural integrity of pVHL (Fig. S3).

The selected variants were mapped into native protein structure using SwissPDB Viewer and energy minimized using Chiron, a rapid protein energy minimization server. The native protein model and mutation-mapped models were aligned using PyMOL, and the extent of structural variation in the mutated models were assessed using RMSD scores (Table S2). The missense variants were ranked based on the RMSD score, their structural importance (presence of missense variant on interactive residue of the α and β domain), conservation with zebrafish VHL protein, and the number of patient reports have been taken as exclusion criteria for selecting the highly potential missense variants (Table S3 and S4, Fig. S4).

Based on the RMSD score, structural importance, and their conservation to zebrafish VHL one SNP position was selected from ElonginC binding domain; C162. The detailed steps in the generation of *vhl*^−/−^ mutant line are represented in Fig. S5. The sgRNA target sequence was designed using the CHOPCHOP online tool.

### Identification of *vhl* mutation by Sanger sequencing

**Fig. 4.**
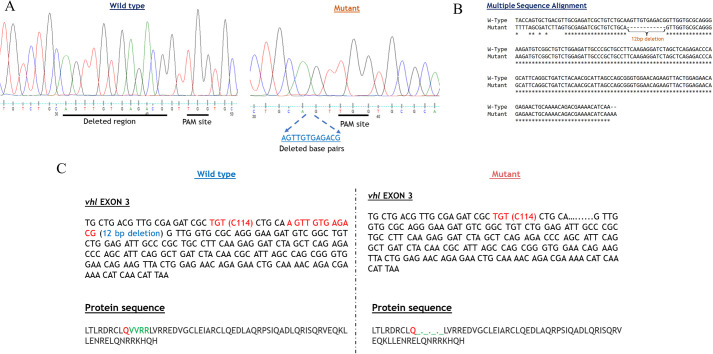
**Identification of target site mutation by Sanger sequencing.** (A) Identification of mutation by Sanger sequencing in wild-type target site sequence, and mutant target site sequence. (B) Multiple sequence alignment of wild-type and mutant target site sequence by CLUSTALW. (C) Comparison of wild-type and mutant exon 3 DNA sequence and their corresponding amino acid sequences.

The target sites were amplified from the F_2_ *vhl* mutants through PCR. Subsequently, the PCR products from the target site were purified and subjected to sequencing (Fig. 4A). The sequencing results revealed the nucleotide sequences of mutant alleles with 12 base pairs deletion at the *vhl* target site (Fig. 4A,B). The 12-base pair deletion identified in the zebrafish *vhl* gene results in the deletion of four amino acids in the corresponding to ElonginC binding domain of the VHL protein (Fig. 4C).

### Characterization of zebrafish *vhl*^−/−^ mutant line

The heterozygous mutants with the known mutation were outcrossed to wild type and *Tg(fli1a:EGFP)^y1^* to generate a large number of F_2_ heterozygous adults with the same mutation. The F_2_ heterozygous zebrafish *vhl* mutants were identified using a heteroduplex mobility assay, which involved running the PCR-amplified target site on a 15% acrylamide gel. Subsequently, the heterozygous mutants from wild type and *Tg(fli1a:EGFP)^y1^* backgrounds were incrossed to produce zebrafish *vhl* mutants with biallelic loss.

Until 4 dpf, the *vhl* mutants with biallelic loss could not be distinguished morphologically from the heterozygous population. However, starting at 4.5 dpf, the *vhl^−/−^* mutants could be differentiated from the heterozygous *vhl^+/−^* larvae by observing their ability to inflate the swim bladder (Fig. S7). The *vhl^−/−^* mutants failed to inflate their swim bladders, clearly distinguishing them from *vhl* heterozygous and wild-type larvae (Fig. S7). This hypoxia phenotype exhibited by the *vhl* mutants corroborates with previous studies (van Rooijen et al., 2009; Santhakumar et al., 2012).

The *vhl^−/−^* mutants, separated based on the absence of swim bladder inflation, were confirmed through genotyping by comparing PCR-amplified target site sizes on a 15% acrylamide gel (examples for differentiating the wild type, hetero and homozygous mutants using this method is given in Fig. 7B).

**Fig. 5.**
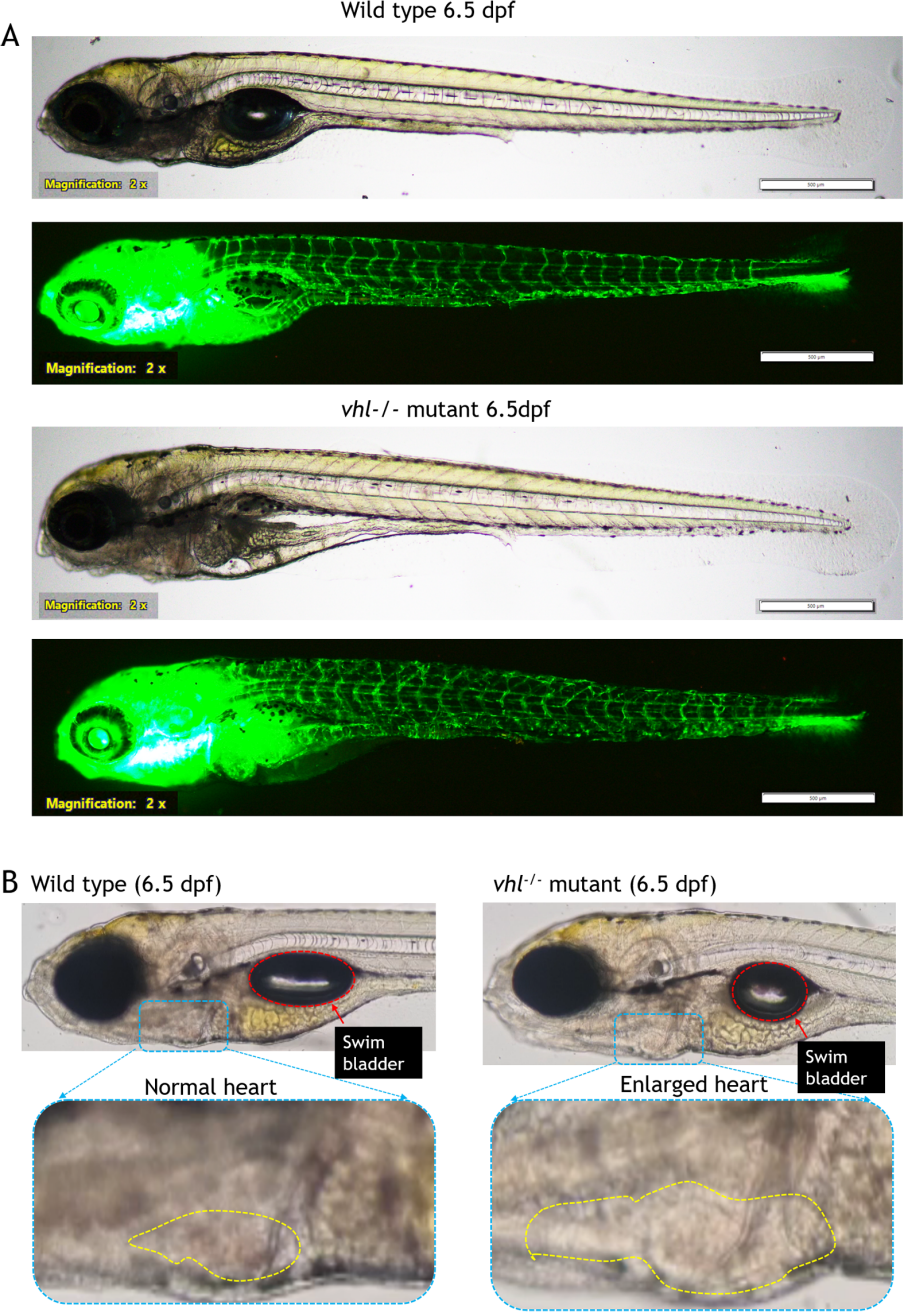
**Analysis of blood vessel morphology and heart size between wild type and *vhl^−/−^* mutant.** (A) Comparison of blood vessel in wild type and *vhl^−/−^* mutant at 6.5 dpf. (B) Comparison of heart size in wild type and *vhl^−/−^* mutant.

**Fig. 6.**
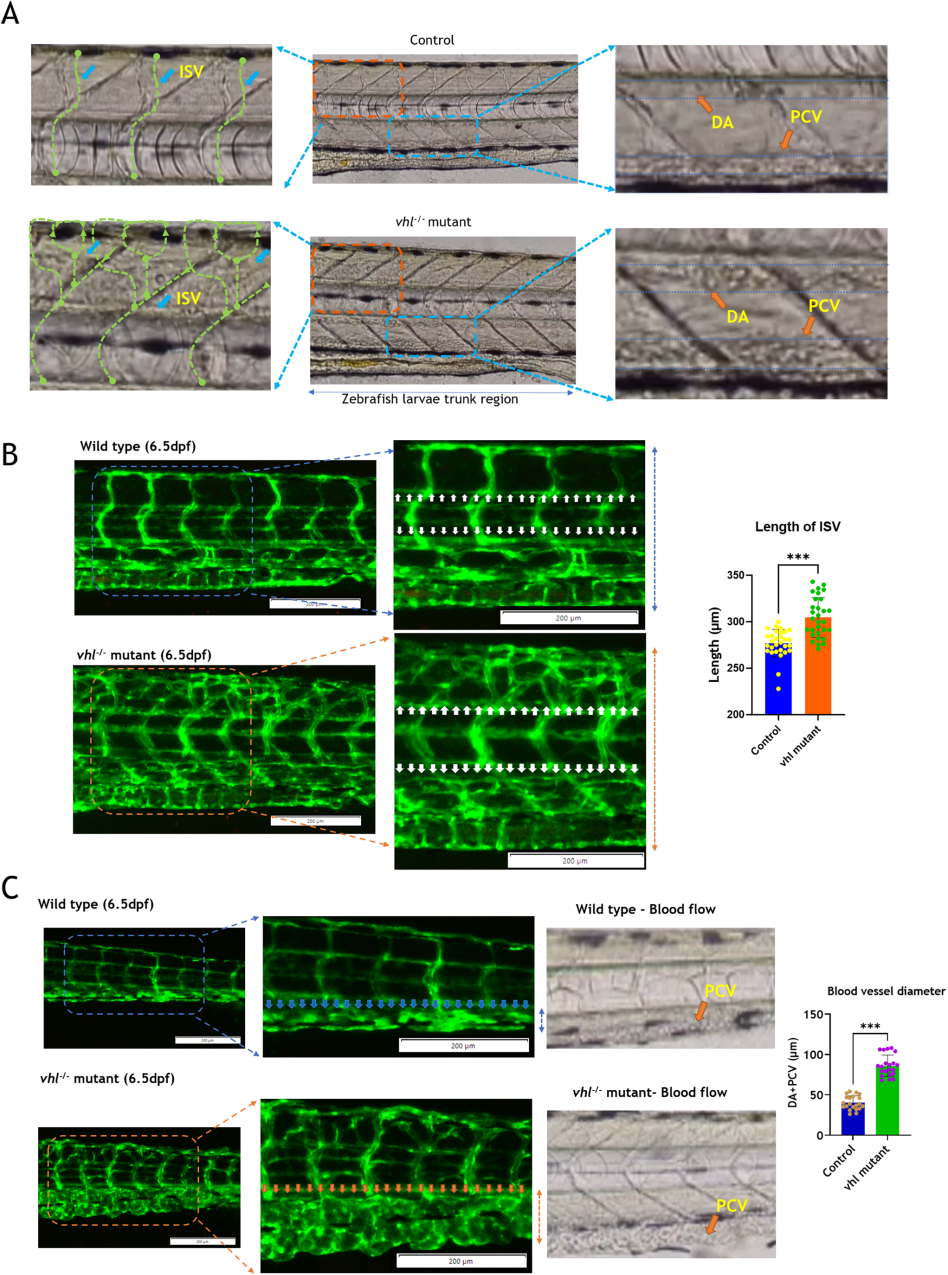
**Analysis of differences in blood flow and blood vessel morphology between wild type and *vhl^−/−^* mutant.** (A) Comparison of blood flow in dorsal aorta (DA), posterior cardinal vein (PCV) and ISV of wild-type and *vhl^−/−^* mutant zebrafish larvae at 6.5 dpf. (B) Comparison of blood vessels in ISV region of (a) wild-type and (b) *vhl^−/−^* mutant zebrafish larvae at 6.5 dpf. (C) Comparison of blood vessel and blood flow in the tail region (DA+PVC) of (a) wild-type and (b) *vhl^−/−^* mutant zebrafish larvae at 6.5 dpf Tg (*fli1a*:*EGFP*). For measurement of ISV length and blood vessels diameter three areas were measured for each larva (the right, left and the middle area of the specified region). Sample number for ISV length=10*3 measurement areas (30 measurements values). Number of samples in blood vessels diameter=7*3 (27 measurement values). Two-tailed unpaired *t*-test. Error bar: Mean±s.d.; ***P≤0.001.

From 4.5 dpf onwards, the mutants exhibited increased blood flow in the intersegmental regions due to neovascularization (Fig. S7). To confirm this, the heterozygous *vhl^+/−^* mutants with the *Tg(fli1a:EGFP)^y1^* background were incrossed, and blood vessels were observed starting at 4.5 dpf (Fig. S7). Observations of *vhl^−/−^* mutants under a fluorescence microscope revealed the presence of newly formed ectopic blood vessel branches in the ISV region (Fig. S7; Fig. 5A). At 6.5 dpf, the *vhl* mutants displayed slight inflation of the swim bladder, an enlarged heart (Fig. 5B; Movie 2), multi-layered blood flow in the dorsal aorta (Fig. 6A; Movie 3), ectopic branching due to neovascularization throughout the body (Fig. 6B), and highly dilated blood vessels with trafficking in the tail region (Fig. 6C; Movie 4), all due to increased and multi-layered blood flow. These observations align closely with the phenotypes of *vhl^−/−^* mutants described in previous studies. Also, as described in the paper by van Rooijen et al. (2009) *vhl^−/−^* mutants survived only till 10-12 dpf. These findings clearly confirm that the generated zebrafish mutant line has lost the function of *vhl*.

**Fig. 7.**
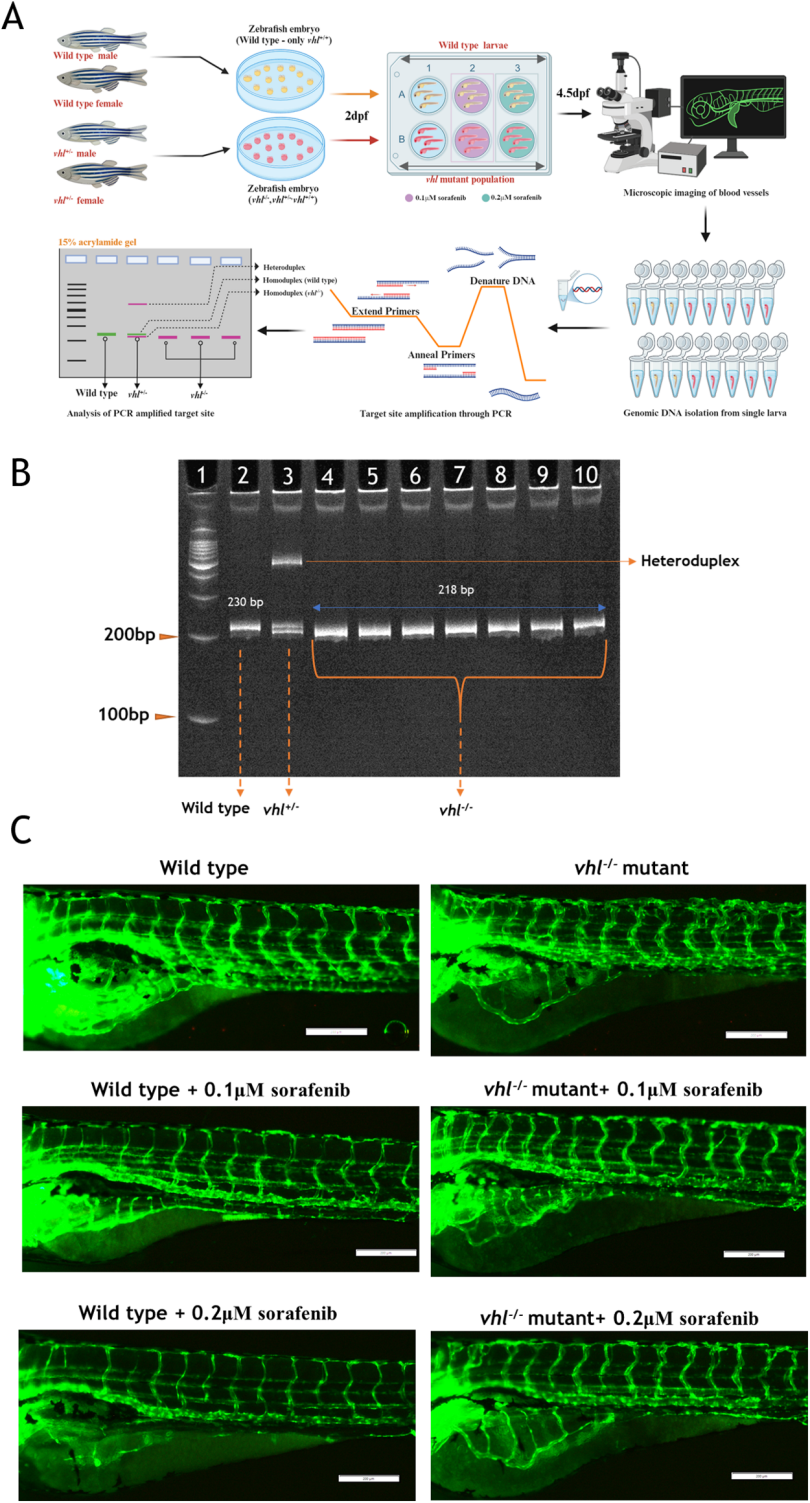
**Effect of sorafenib on *vhl^+/−^* mutant zebrafish larvae.** (A) Steps in differentiation of *vhl^+/−^*, *vhl*^+/−^ and *vhl*^−/−^ from mixed VHL mutant zebrafish larvae population after sorafenib treatment. (B) Differentiation of wild-type, *vhl^+/−^* and *vhl^−/−^* genotypes using target site amplified PCR products analyzed on a 15% acrylamide gel. (C) Images showing changes in SIV and ISV regions of zebrafish larvae *Tg(fli1a*:*EGFP)* at 4.5 dpf. Created in BioRender by Chakraborty, P., 2025. https://BioRender.com/o94q590. This figure was sublicensed under CC-BY 4.0 terms.

### Identifying the effect of antiangiogenic drug in *vhl^−/−^* mutant

To further investigate our hypothesis on the efficacy of sorafenib treatment under hypoxia, we assessed the effects in the genetically induced hypoxic *vhl*^−/−^ zebrafish mutant model by treating with the antiangiogenic drug, sorafenib. A challenge in conducting this experiment was distinguishing the *vhl*^−/−^ mutants from the siblings (*vhl*^+/+^, *vhl*^+/−^, and *vhl*^−/−^).

To address this, we followed a systematic approach outlined in the Fig. 7A. The larvae were exposed blindly to sorafenib and subjected to post-hoc genotyping (Fig. 7B). Subsequently, we presented blood vessel images for all groups: control, control+0.1 µM sorafenib, control+0.2 µM sorafenib, *vhl*^−/−^+0.1 µM sorafenib, and *vhl*^−/−^+0.2 µM sorafenib (Fig. 7C).

**Fig. 8.**
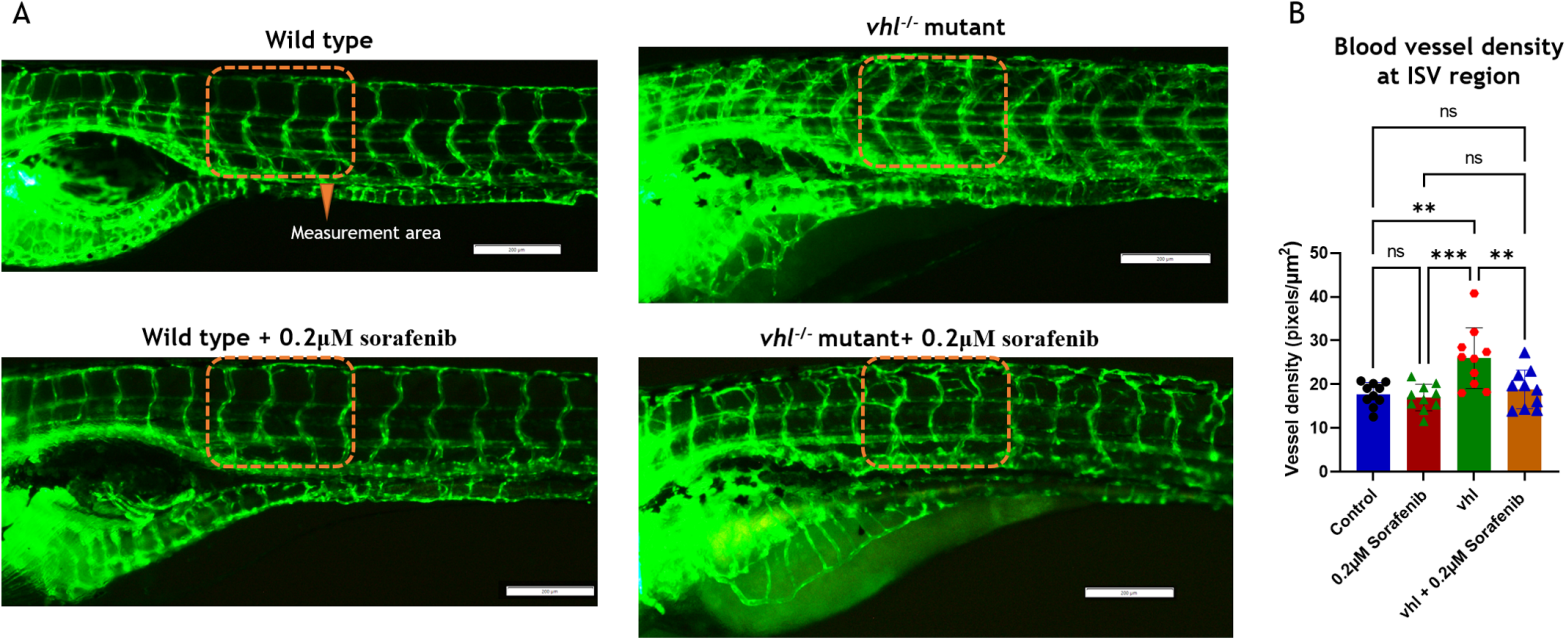
**Effect of sorafenib on genetic pseudo-hypoxia zebrafish model.** (A) Images showing effect of sorafenib in SIV and ISV regions of wild-type and *vhl^−/−^* mutant zebrafish larvae *Tg(fli1a*:*EGFP)* at 6.5 dpf. (B) Average blood vessel density in ISV region (*n*=10). One-way ANOVA, Tukey's post-hoc test. Error bar: Mean±s.d.; **P≤0.01, ***P≤0.001; ns, non-significant.

**Fig. 9.**
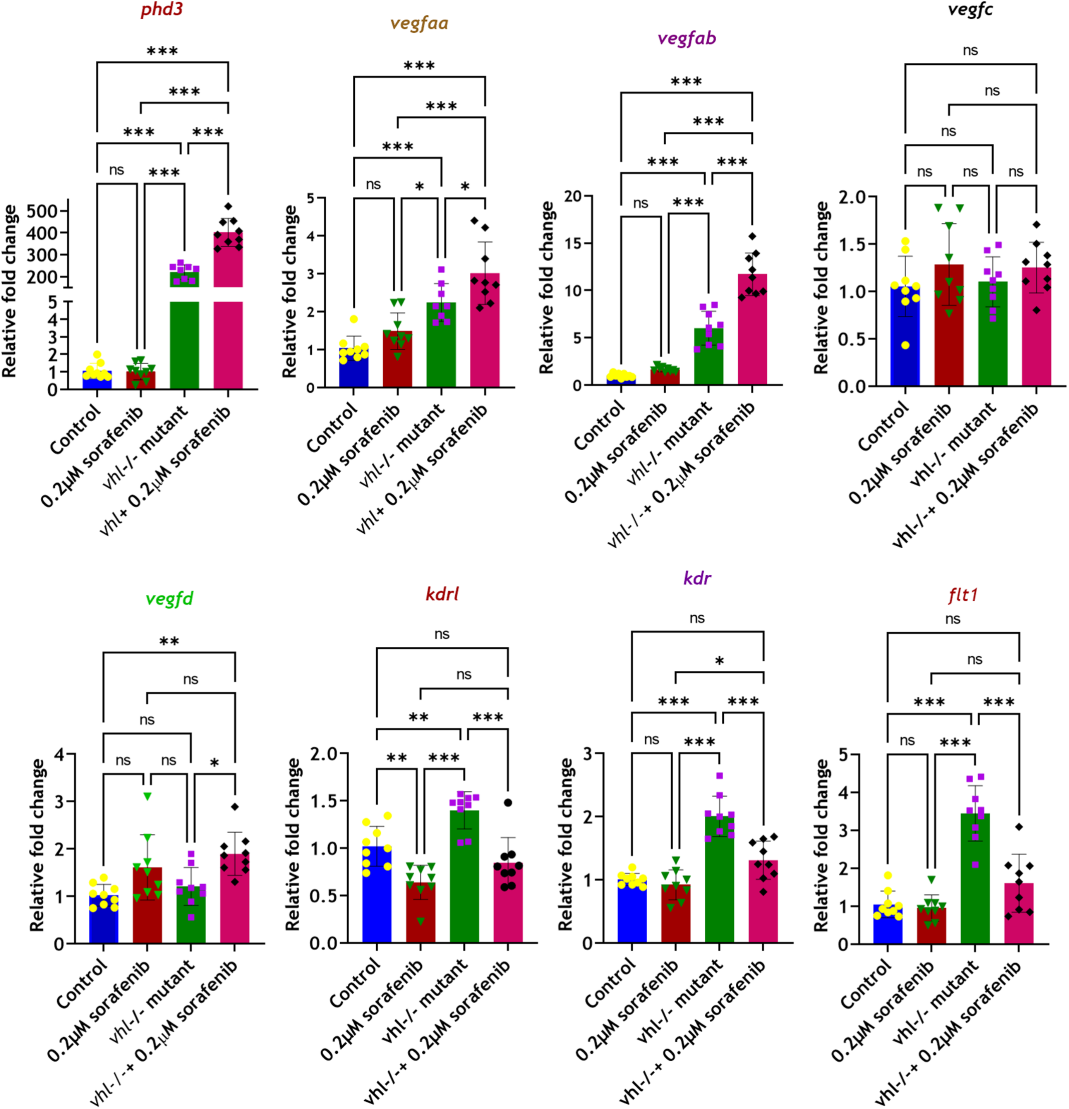
**Comparative gene expression analysis by RT-qPCR.** 50 larvae per each well, biological triplicate for each group, for each biological triplicate, three technical triplicates were kept in RT PCR. *n*=biological triplicate*technical triplicate=3*3=9values for each group. One-way ANOVA, Tukey's post-hoc test, error bar: Mean±s.d.; *P≤0.05, **P≤0.01, ***P≤0.001; ns, non-significant.

To accurately assess gene expression, a large number of *vhl*^−/−^ mutant larvae were required. These mutants were easily identifiable by the absence of a swim bladder and increased blood flow in the ISV due to ectopic neovascularization. Thus, sorafenib treatment for the *vhl*^−/−^ group was conducted from 5 dpf and continued till 7 dpf. At 7 dpf, we captured blood vessel images for all groups (control, control+0.1 µM, control+0.2 µM, *vhl*^−/−^+0.2 µM sorafenib) (Fig. 8). Gene expression studies were then performed at this stage using RT-qPCR for all groups (Fig. 9). We opted to use *gapdh* as our endogenous control instead of beta-*actin*, given the variation in expression levels observed in *vhl^−/−^* mutants.

When comparing the effects of sorafenib on the genetic zebrafish *vhl^−/−^* hypoxia model, we found results consistent with those observed in the DMOG-induced chemical pseudo-hypoxia model. Compared to control and *vhl^−/−^* mutants, the *vhl*^−/−^ mutants exhibited widespread ectopic neovascularization, particularly noticeable in the ISV, where irregularly patterned blood vessel capillaries formed (Figs 7C and 8). In contrast, the control group displayed regularly spaced ISV with clear gaps in between, as illustrated in the accompanying Fig. 7C (4.5 dpf) and Fig. 8 (7 dpf).

Sorafenib treatment of wild-type larvae completely inhibited vessel establishment in SIV region (Fig. 7C), while the SIV region remained entirely protected in the *vhl^−/−^* mutants upon sorafenib treatment (Figs 7C and 8). Notably, the effects of sorafenib were evident in the ISV region of *vhl^−/−^* mutants, where ectopic neovascularization was inhibited, resulting in a regularly structured ISV similar to that seen in wild-type larvae in both 0.1 μM and 0.2 μM concentrations at 4.5 (Fig. 7C) and 7 dpf stages (Fig. 8A,B).

The RT-qPCR analysis revealed an increase in cellular hypoxia signaling levels in the *vhl^−/−^* model following sorafenib treatment, mirroring the patterns observed in the chemical pseudo-hypoxia model. The average expression of *phd3*, a hypoxia marker gene in zebrafish, was 220-fold higher in *vhl* mutants compared to controls. Following sorafenib treatment, this level increased to 401-fold, reflecting a similar response to that observed in the chemical pseudo-hypoxia model (Fig. 9). Additionally, proangiogenic factors such as *vegfaa* and *vegfab* showed significant increases in expression levels in *vhl*^−/−^ mutant, while *vegfc* exhibited no change in expression (Fig. 9). Conversely, *vegfd* showed a significant increase only when the *vhl*^−/−^ mutants were treated with sorafenib (Fig. 9). The expression level of *vegfd* in *vhl^−/−^* knockout mutants remained unchanged compared to wild type, suggesting that *vegfd* may play a key role in mitigating the effects of sorafenib (Fig. 9).

In the *vhl^−/−^* mutants, the expression levels of *vegf* receptors (*kdrl*, *kdr*, *flt1*) were significantly elevated, but these levels were notably downregulated upon sorafenib co-treatment (Fig. 9). This downregulation likely contributes to the inhibition of ectopic branching (terminal branches) in the ISV of *vhl* mutant zebrafish larvae (Figs 7C and 8). The increase in cellular hypoxia levels, along with the elevated expression of *vegfaa*, *vegfab*, and *vegfd* during sorafenib treatment, helped protect the primary and secondary branches of SIV and ISV region (Figs 7C and 8). Consequently, both the SIV and ISV vessels were completely protected from sorafenib treatment, while terminal neovascularization in the ISV was significantly inhibited, as clearly shown in Fig. 7C.

## DISCUSSION

Zebrafish, with its transparent early larval stages, serves as an excellent model for anti-angiogenic drug testing. The antiangiogenic efficacy of a drug is routinely assessed by treating the wild-type zebrafish larvae. So, we hypothesized that while a drug might show promise under normal conditions in zebrafish, its efficacy could differ significantly when applied to activated hypoxia signaling conditions. To validate this hypothesis, we generated two pseudo-hypoxia models in zebrafish: (1) chemical pseudo-hypoxia model by inhibiting the PHD enzymes using DMOG, and (2) a genetic pseudo-hypoxia model by *vhl* gene knockout via CRISPR mutagenesis.

Our initial aim was to closely examine the area where pro-angiogenesis occurs in the zebrafish larval blood vessels after hypoxia signaling activation in both chemical and genetic pseudo-hypoxia models. Following this, we planned to test our hypothesis by treating both normal and pseudo-hypoxia zebrafish larvae with sorafenib to evaluate the compound's effects under these distinct conditions.

### Identification of pro-angiogenic area in pseudo-hypoxia models

Firstly, we developed DMOG induced pseudo-hypoxia model in zebrafish larvae and observed the early angiogenic pattern in sub intestinal vessel region at 3 dpf as similar to 4 dpf control. Then it was identified that DMOG causes some changes in vessel pattern over the region of liver. We started observing the blood vessel network from 4 dpf zebrafish larvae using *O-*dianisidine staining. By implementing this method, we observed the blood vessel development pattern in the liver from 4 to 6 dpf zebrafish larvae. Then we compared the liver angiogenic pattern of control and treated zebrafish larvae and found DMOG-treated larvae showed a significant increase in the liver angiogenic area.

One important point to emphasize is that the *O*-dianisidine staining method is specifically useful for observing liver angiogenic patterns. However, in the case of *vhl* mutants, the changes occur in the SIV and ISV (intersegmental vessel) regions, which are not captured by this staining method. Due to these limitations, we have used *Tg(fli1a*:*EGFP)^y1^* for further experiments.

We next developed a genetic pseudo-hypoxia model using a CRISPR mutagenesis approach and generated a *vhl* mutant line, which induced a 12-base pair deletion in the ElonginC binding domain. Unlike the DMOG model, the pro-angiogenesis in *vhl^−/−^* can be seen through the complete zebrafish larva from 4 dpf onwards (Van Rooijen et al., 2009). From this stage the terminal ectopic neovascularization was observed throughout the body especially between the ISV region but in the DMOG treated larvae had no such terminal neovascularization in ISV region. This clear-cut evidence of pro-angiogenesis in *vhl*^−/−^ mutants indicated it is a superior model and was therefore used in the later work.

### Identifying the effect of antiangiogenic drug on pseudo-hypoxia models

When we treated with 0.1 μM and 0.2 μM sorafenib in the chemical pseudo-hypoxia model. At lower concentrations it increased angiogenesis in SIV region. In the 0.2 μM sorafenib treated group, dilation and incomplete inhibition of SIV region was observed. This is a clear indication that the DMOG-treated larvae were suppressing the effect of the sorafenib.

Consistent with the DMOG model, the *vhl*^−/−^ mutants also showed resistance against sorafenib where it completely protected the SIV and ISV even at 0.2 μM treatment of sorafenib. But the effect of sorafenib can be observed even at lower concentration sorafenib (0.1 μM) using *vhl*^−/−^ mutants. As it completely inhibits neovascularization in the ISV region (whereas in the DMOG model there was no terminal neovascularization), it does not make an ideal model for identifying the antiangiogenic properties of the compound at a lower concentration.

Gene expression studies indicated a drastic increase in the expression level of HIF signaling activation marker gene, *phd3*, in DMOG and sorafenib co-treatment group. We also found that the expression levels of pro-angiogenic factors, such as *vegfaa* and *vegfab*, were increased in the DMOG and sorafenib co-treated group. But VEGF receptors, such as *flt1*, *kdr*, and *kdrl*, showed no significant increase in expression in DMOG, or DMOG and sorafenib co-treatment group. Sorafenib efficiently downregulated the expression levels of *flt1*, *kdr* and *kdrl*, as expected*.*

In the *vhl* pseudo-hypoxia model, the expression levels of *phd3*, *vegfaa*, and *vegfab* were also significantly elevated when subjected to sorafenib treatment. In addition, *vegfd* was only significantly elevated in *vhl*^−/−^ mutants treated with sorafenib. These factors suggest the reasons behind protection of the secondary vessels SIV and ISV in pseudo-hypoxia models upon sorafenib treatment. In addition VHL mutants displayed significantly elevated expression levels of VEGF receptors such *kdr*, *kdrl*, and *flt1*, and interestingly their expression levels were observed to be downregulated to normal levels upon sorafenib treatment. This could be the potential reason behind the elimination of terminal branches between ISVs in sorafenib-treated *vhl* mutants.

In the *vhl* pseudo-hypoxia model, the expression level of *phd3*, *vegfaa*, and *vegfab* were significantly elevated and their expression levels further enhanced when larvae were subjected to sorafenib treatment. In addition, *vegfd* was observed to be significantly increased only in *vhl*^−/−^ mutants when subjected to sorafenib treatment. These elevated VEGF levels might trigger the secondary vessels SIV and ISV in pseudo-hypoxia models from sorafenib treatment. In addition, VHL mutants displayed significantly elevated expression levels of VEGF receptors such *kdr*, *kdrl*, and *flt1*, which were brought back to normal levels when subjected to sorafenib treatment. This could be the reason behind the elimination terminal branches between ISV. In conclusion, our results support our hypothesis that the effect of an antiangiogenic drug can vary when subjected to activated hypoxia signaling conditions, compared to normal conditions.

The *vhl^−/−^* model is particularly well-suited for testing antiangiogenic drugs over the DMOG-mediated hypoxia model, because the chemical model exhibits very low activation of hypoxia signaling compared to the *vhl^−/−^* model, and importantly, the DMOG-treated larvae did not exhibit the neovascularization phenotype. The genetic *vhl^−/−^* model consistently yields reproducible phenotypes and stable activation of hypoxia signaling, making it an ideal tool for anti-angiogenic drug testing. The effect of sorafenib treatment on *phd3* expression is surprising, which indicates that sorafenib may lead to activation of the HIF/hypoxic signaling pathway. There could be two explanations: the first is that its effect on the vasculature creates a hypoxic environment and therefore leads to activation. An issue with this idea is that zebrafish larvae are very small and it is thought that normal diffusion is generally sufficient to supply oxygen (Pelster, 2008; Rombough and Drader, 2009). Therefore, an alternative explanation might be that the reduction in VEGF signaling as a result of sorafenib treatment might more directly lead to activation of HIF signaling, although this requires further investigation.

## Conclusion

In this study we showed that the efficacy of anti-angiogenic drug activity significantly differs under chemically and genetically mediated pseudo-hypoxic conditions. After observing the effects of sorafenib in *vhl^−/−^*, we have drawn two main conclusions: (1) the *vhl*^−/−^ mutants are highly effective for screening angiogenic drugs, as the effects of sorafenib were distinctly observable in the ISV region even at low concentrations (0.1 μM), providing a clearer visual understanding than the chemical pseudo-hypoxia model. (2) The protective role of the SIV region in *vhl^−/−^* mutants during sorafenib treatment is an important factor to consider in drug screening. Because the complete protection of the SIV and ISV region in *vhl*^−/−^ mutants during sorafenib treatment, compared to the vulnerability observed in control larvae, indicates that increased cellular hypoxia levels and proangiogenic factors such as *vegfaa*, *vegfab*, and *vegfd* contribute to this resistance. Thus, the *vhl*^−/−^ mutant line could serve as a valuable proangiogenic model for efficient testing of anti-angiogenic drugs in cancer therapeutics.

## MATERIALS AND METHODS

### Zebrafish maintenance

The zebrafish wild-type and *Tg(fli1a*:*EGFP)^y1^* lines were housed in a Tecniplast^®^ recirculating zebrafish system, maintained under a 14-h light and 10-h dark cycle at 28.5°C. For breeding, males and females were crossed in a 2:1 ratio. The embryos were grown under controlled conditions at 28.5°C±0.5°C, adhering to standard protocols (Kimmel et al., 1995). Subsequently, they were utilized for the experiments as detailed below.

### Chemical compound treatments

Zebrafish larvae (*n*=10) were treated with 100 μM of DMOG solution [dissolved in 0.1% dimethyl sulfoxide (DMSO)] for DMOG treatment, whereas the control embryo medium had only 0.1% DMSO (van Rooijen et al., 2009). Treatment was started in 2 dpf zebrafish larvae and continued up to 4 dpf with the refreshment of a new medium and DMOG each day. The same procedure was followed for 0.1 and 0.2 μM sorafenib treatments, either separately or in co-treatment mode along with 100 μM DMOG.

### Blood vessel observation by ImageJ

To aid blood vessel observation, zebrafish larvae were sedated with 0.835 µM of MS222 (Machikhin et al., 2020). Blood flow video was recorded from 1 to 4 dpf zebrafish larvae using a compound microscope, and images/videos were captured with a cell phone camera. The recorded video files were processed into separate image sequences. The original images were then converted to grayscale. These images were imported to ImageJ for processing, where the vascular patterns were visualized using an image subtraction (Vinoth et al., 2022).

### *O*-dianisidine staining

The zebrafish larvae were transferred to 2 ml microcentrifuge tubes containing 0.168 mg/ml tricaine. Once the zebrafish larvae were anesthetized, the solution was removed, and the larvae were washed with PBST. Then the larvae were added into the staining solution containing 0.6 mg/ml *O*-dianisidine, 20 μl/ml H_2_O_2_, 10 mM sodium acetate (pH 5.2), and 40% ethyl alcohol. After adding the staining solution, the zebrafish larvae were incubated in the staining solution for 20 min at room temperature. Then the larvae were washed three times with phosphate buffered saline with tween 20 (PBST) and fixed in 4% formaldehyde solution at 4°C overnight. Then the larvae were bleached with a bleaching solution containing 0.9% sodium hydroxide solution, 0.8% potassium hydroxide, and 0.1% tween 20. The larvae were then incubated at room temperature for 40 min. Following this the bleaching solution was removed, and the larvae were washed with PBST three times. Finally, the larvae were stored at 4°C with the addition of 1 ml PBS (Detrich et al., 1995).

### Microscopic imaging

Microscopic images were captured using an Olympus BX53 upright fluorescent microscope using cellSens software. After acquiring the images, they were processed in Microsoft PowerPoint. This involved cropping the images to focus on relevant areas of interest and arranging them into cohesive groups. Standard scale bars provided by cellSens software were incorporated into each image to maintain consistency and to facilitate accurate size estimation across different microscopic views.

### RNA extraction

RNA extraction from zebrafish larvae was performed using the TRIzol method. Each experimental group consisted of 50 larvae, with three replicates per group. The larvae were homogenized in 700 μl of Trizol reagent to facilitate cellular disruption and RNA extraction. Following homogenization, 200 μl of chloroform was added, and the tubes were gently inverted to ensure thorough mixing. Subsequently, the samples were centrifuged at 12,000 rpm for 15 min to separate them into aqueous and organic phases. The aqueous phase, containing RNA, was carefully transferred to new 1.5 ml microcentrifuge tubes. To precipitate RNA, an equal volume of isopropanol was added and gently mixed. After centrifuging at 12,000 rpm for 5 min, the supernatant was discarded, and the RNA pellet was washed twice with 1 ml of 75% ethanol. Each wash involved centrifugation at 12,000 rpm for 5 min. Finally, the RNA pellet was air-dried and dissolved in 30 μl of Milli-Q water. The concentration and purity of RNA were assessed using a Nanodrop spectrophotometer, and RNA integrity was confirmed by running samples on a 2% agarose gel (Peterson and Freeman, 2009).

### Reverse transcription

The reverse transcription of RNA to complementary DNA (cDNA) was conducted using the iScript™ cDNA Synthesis Kit from Bio-Rad, following the manufacturer's protocol. Briefly, 1 μg of total RNA was added to a reaction tube containing reaction mix, reverse transcriptase enzyme, buffer, and the volume was adjusted to 20 μl with nuclease-free water. The reaction proceeded with priming at 25°C for 5 min, followed by reverse transcription at 46°C for 20 min. Finally, the enzyme was inactivated by heating to 95°C for 1 min.

### Real time quantitative PCR (RTqPCR)

RTqPCR was conducted to quantify the expression levels of the target gene using Applied biosystems QuantStudio 1. Primers specific to the target gene and reference gene were designed using Primer-BLAST. The RTqPCR reaction mixture consisted of 5 μl of Applied Biosystems™ PowerUp™ SYBR™ Green Master Mix, required volume of forward and reverse gene specific primers, 1 μl of 1:25 diluted cDNA and the volume was made up to 10 μl using nuclease free water. Cycling conditions were as follows: initial denaturation at 50°C for 2 min, 95°C for 10 min, followed by 40 cycles of denaturation at 95°C for 15 s, annealing at 60°C for 1 min. Fluorescence data were collected during the annealing step of each cycle. Quantification cycle (Cq) values were determined using QuantStudio™ Design and Analysis Software v1.5.2 and analyzed using the comparative ΔΔCt method. Results were normalized to the expression levels of the reference gene β-actin and presented as fold change relative to control group. Statistical analysis was performed using GraphPad.Prism.9.0.0.121 and significance was set at *P*<0.05. To validate the specificity of the qPCR products, melting curve analysis was performed at the end of each qPCR run. All experiments were performed in triplicate to ensure reproducibility of results (Lan et al., 2009). The details of primers used in gene expression analysis were given in Table S1.

### Acquisition of *VHL* missense variant dataset and retrieval of VHL protein structure

The non-synonymous single nucleotide polymorphisms (nsSNPs) data and the full-length protein sequence of VHL were gathered from the Ensembl database. The 3D structure of VHL protein (PDB ID:1VCB), derived from X-ray crystallography was obtained from the PDB (Kouranov et al., 2006).

### Screening deleterious nsSNPs

The study utilized a range of tools to predict the deleterious effects of nsSNPs. The SIFT algorithm, developed by Kumar et al. (2009), leverages sequence homology to assess how amino acid substitutions may impact protein function, categorizing changes as deleterious or tolerated (Kumar et al., 2009). PolyPhen-2, another tool cited, predicts structural and functional alterations induced by amino acid changes using evolutionary conservation and protein structure data (Adzhubei et al., 2013). PANTHER (protein analysis through evolutionary relationships) identifies evolutionary conservation timelines of amino acids and predicts their impact on protein structure and function (Mi et al., 2016). PROVEAN, as referenced, assesses the biological function of proteins affected by amino acid changes (Choi and Chan, 2015). PMUT annotates and predicts pathological nsSNPs, while SNP&GO predicts disease-associated nsSNPs (Calabrese et al., 2009). Align-GVGD combines amino acid biophysical characteristics and multiple sequence alignments to predict the impact of missense substitutions (Tavtigian et al., 2008). MutPred2 classifies amino acid substitutions as disease-associated or neutral, also predicting the molecular causes of disease (Pejaver et al., 2020). Each tool was provided with the protein sequence, UniProt ID, and PDB ID of both wild-type and mutant residues of VHL as input. Outputs, including scores and predictions, were recorded. nsSNPs identified as deleterious across all analyses underwent further assessment for protein stability.

### Assessing the impact of deleterious nsSNPs in protein stability

All deleterious nsSNPs identified in the previous analysis underwent further scrutiny to confirm their impact on protein stability using the following tools: mCSM: This tool relies on graphical-based signatures to assess mutation impact by correlating atomic-distance patterns around amino acid residues. It predicts changes in protein stability, as well as affinity for protein-protein and protein-deoxyribonucleic acid (DNA) complexes (Pires et al., 2014). MUpro predicts changes in protein stability for single-site nsSNPs solely from sequence information, overcoming limitations of methods requiring tertiary structure data (Cheng et al., 2006). iMUTANT is a neural-network-based web server for automatic prediction of protein stability changes due to single-site mutations (Capriotti et al., 2005). iSTABLE is available with structural and sequential inputs, this tool integrates predictions for protein stability changes upon single mutations (Chen et al., 2013). The web server Site Directed Mutator (SDM) employs conformationally constrained environment-specific substitution tables (ESSTs) to compute stability differences between wild-type and mutant protein structures, also predicting potential structural impacts in disease (Pandurangan et al., 2017). CUPSAT predicts changes in protein stability caused by point nsSNPs using amino acid-atom potentials and torsion angle distribution, while considering solvent accessibility and secondary structure specificity (Parthiban et al., 2006). Following analysis, the tools provided scores for changes in protein stability (ΔΔG scores) and assessed the stability change status for the identified deleterious nsSNPs.

### Incorporating nsSNPs into native protein structure and analysis of structural deviation in mutated protein models

The nsSNPs identified in our analyses were mapped onto the native protein structure using SwissPDB Viewer. Subsequently, the mutated models underwent energy optimization using Chiron, a rapid protein energy minimization server. The native protein model and mutation-mapped models were aligned using PyMOL, and the extent of structural variation in the mutated models was assessed using RMSD (root-mean-square deviation) scores. Additionally, clinically reported mutations were obtained from the VHL database (VHLdb) (http://vhldb.bio.unipd.it/home), and our results were compared with those already reported (Minervini et al., 2019; Tabaro et al., 2016).

### Design and synthesis of sgRNA template

The sgRNA target sequence was designed using the CHOPCHOP online tool (https://chopchop.cbu.uib.no/). The T7 promoter region was added to the target sequence along with the over lapping region of constant oligonucleotide (Fig. S6). The target sequence and the constant oligonucleotides were synthesized from Bioserve Biotechnologies India Pvt Ltd. The sgRNA template was synthesized using following method 1 μl of gene-specific oligo (100 μM), 1 μl of constant oligo (100 μM), 5 μl of amplicon red PCR master mix, and the total volume was made up to 20 μl with MilliQ. The sgRNA template was synthesized by keeping the reaction mix by following the PCR reaction protocol consisting of initial denaturation at 95°C for 5 min, followed by 30 cycles of denaturation at 95°C for 1 min, annealing at 45°C for 30 s, and extension at 72°C for 1 min, with a final extension step at 72°C for 5 min, and then held at 4°C (Gagnon et al., 2014).

### Synthesis of sgRNA by *in vitro* transcription

The sgRNA was synthesized by *in vitro* transcription using the HiScribe^®^ T7 High Yield RNA Synthesis Kit. The reaction mix included 1 µg of sgRNA template, 1.5 µl each of ribonucleotide triphosphates (rNTPs) (rATP, rGTP, rCTP, rUTP) (10 mM), 2 µl of T7 RNA polymerase, 1.5 µl of reaction buffer, and the total volume was adjusted to 20 µl using nuclease-free water. The reaction mix was then incubated at 37°C for 2 h.

Following *in vitro* transcription, the reaction mix underwent DNase treatment. A 20 µl sample was mixed with 17.5 µl of 10X buffer, 5 µl of DNase I enzyme (1 U/µl) from Thermo Fisher Scientific, and 150 µl of nuclease-free water. The mixture was incubated at 37°C for 15 min.

After DNase treatment, the reaction mixture was purified using the Monarch NEB RNA Purification Kit. A 200 µl sample was combined with 400 µl of Monarch Binding Buffer and an equal volume (600 µl) of ethanol, mixed well at room temperature, added to a column, and centrifuged at 15,000 rpm for 1 min. The flow-through was discarded, and the column was washed twice with 500 µl of wash buffer, each time centrifuged at 15,000 rpm for 1 min. Next, 30 µl of nuclease-free water was added to the column, incubated at room temperature for 1 min, and centrifuged at 15,000 rpm for 1 min into a new 1.5 ml microcentrifuge tube. The RNA was quantified using a Nanodrop spectrophotometer. The quality of sgRNA was assessed by running it on a 2% agarose gel. Finally, the sgRNA was aliquoted, and stored at −80°C.

### Synthesis of Cas9 mRNA

The *pCS2-nCas9n* plasmid (#47929) encoding nCas9n was obtained from Addgene as a bacterial stab. Pure colonies were obtained by streaking on Luria-Bertani (LB) agar plates supplemented with ampicillin (100 μg/ml). These colonies were then inoculated into LB broth containing ampicillin (100 μg/ml). Plasmid DNA was subsequently isolated from the grown bacterial culture (Feliciello and Chinali, 1993). To linearize the pCS2-nCas9n plasmid for Cas9 mRNA synthesis, an 8 μl aliquot of the plasmid (500 ng/μl concentration) was mixed with 5 μl of buffer 3.1 (NEB) and 3 μl of Not I restriction enzyme, bringing the total volume to 50 μl. The reaction mixture was then incubated at 37°C for 2.5 h. Subsequently, 2 μl of the reaction product was analyzed on a 0.8% agarose gel to confirm complete linearization of the pCS2-nCas9n plasmid DNA. The synthesis of Cas9 mRNA using the Invitrogen™ mMESSAGE mMACHINE™ SP6 Transcription Kit involved several key steps. Initially, 6 μl of linearized *pCS2-nCas9n* plasmid (100 ng/μl concentration) was combined with 10 μl of 2X NTP cap mix, 2 μl of 10X buffer, and 2 μl of 10X SP6 enzyme mix, making a total reaction volume. This mixture was then incubated at 37°C for 4 h to facilitate transcription. Following incubation, the transcribed RNA was treated with DNase to remove any residual DNA contamination and subsequently purified, following the same procedure outlined for sgRNA synthesis. This process ensures the production of pure Cas9 mRNA suitable for downstream applications in genetic manipulation (Gagnon et al., 2014).

### Microinjection

To prepare for microinjection, the sgRNA and Cas9 mRNA were mixed in a precise ratio of 1:2, resulting in a final concentration of 300 ng/μl for sgRNA and 600 ng/μl for Cas9 mRNA. In addition to the genetic components, 0.5 μl of Phenol Red per 5 μl sample was added as a vital tracking dye, crucial for visually confirming successful injections under the microscope (Gagnon et al., 2014). Prior to microinjection, male and female zebrafish were carefully paired in the ratio of 2:1 to facilitate spawning. During the microinjection process, the RNA mixture was delicately injected into the yolk of each embryo using a specialized microinjection needle (Vejnar et al., 2016). This technique ensures precise delivery of the nucleic acid into the developing cells, laying the foundation for targeted genetic modifications. Following microinjection, the embryos were gently transferred to 1X E3 solution, an optimized growth medium for zebrafish embryo development. The embryos were then incubated in a BOD incubator set at a stable temperature of 28.5°C, providing an ideal environment for their growth and development (Kimmel et al., 1995).

### Heteroduplex mobility assay

The amplified PCR products, including samples from CRISPR injected zebrafish, were run on a 15% acrylamide gel. The gel was made by mixing 6 ml of 30% acrylamide/Bis-acrylamide solution (29:1 ratio), 2.4 ml of 5X TAE buffer, 3.6 ml of MilliQ water, 60 µl of 10% ammonium persulfate (APS) as the initiator, and 12 µl of tetramethylethylenediamine (TEMED) as the catalyst. After loading the samples into the gel wells, electrophoresis was conducted at 200 volts (V) for 90 min. Following electrophoresis, the gel was immersed in a 0.5 µg/ml ethidium bromide solution and placed on a gel rocker for 1 min to stain the DNA. Subsequently, the gel was visualized under UV light using a gel documentation unit to detect the presence of heteroduplexes, indicative of successful genome editing in the CRISPR complex-injected samples (Vanleuven et al., 2018).

### Zebrafish outcrossing and identification of heteroduplex positives

After confirming the gene knockout, zebrafish larvae form the knockout positive group were reared in a Tecniplast® zebrafish housing recirculation system. Subsequently, they were crossed with wild-type/*Tg(fli1a*:*EGFP)^y1^* zebrafish to generate heterozygous mutants. The embryos were cultured at 28.5°C in E3 medium using a BOD incubator. At the 2 dpf stage, genomic DNA was isolated from individual larvae, and the target site was amplified and subjected to a heteroduplex assay (Vanleuven et al., 2018). The presence of heteroduplexes indicated successful germline transmission of the mutation to the F_1_ progeny. after that F_1_ heterozygous mutants were crossed to obtain homozygous *vhl^−/−^* mutant.

### PCR product purification

The target site was amplified from F_2_ *vhl^−/−^* genomic DNA, and the resulting PCR product was purified using the Qiagen QIAquick® PCR purification kit. During purification, all centrifugations were conducted at 13,000 rpm at room temperature. To enhance purification efficiency, a pH indicator was added to buffer PB at a ratio of 1:250. Initially, 400 µl of binding buffer was mixed with 80 µl of the PCR product from the target site. This mixture was then transferred to a 1.5 ml column and centrifuged for 60 s. Subsequently, 750 µl of washing buffer (Buffer PE) was added to the column and centrifuged again for 60 s. After discarding the flow-through, the column was centrifuged for an additional minute to remove any residual buffer. The purified DNA was then eluted by adding 50 µl of EB to the column, followed by centrifugation for 1 min. The quantity of the purified PCR product was assessed using a Nanodrop spectrophotometer, and its quality was confirmed by running an analysis on a 2% agarose gel. Then the amplified and purified target site was sequenced, and the mutation was identified using sanger sequencing.

### Steps in identification of the effect of chemotherapy in *vhl^−/−^* mutant

Initially, *vhl^−/−^* mutant larvae with *the Tg(fli1a:EGFP)^y1^* background were treated with 0.1 and 0.2 μM sorafenib from 2 dpf to 4.5 dpf. At 4.5 dpf, images of the blood vessels were captured, and the individual larvae were subjected to genomic DNA isolation. The target site was then amplified, allowing differentiation of the *vhl^+/+^*, *vhl^+/−^*, and *vhl^−/−^* mutants using a heteroduplex mobility assay, which involved running the PCR-amplified target sites on a 15% acrylamide gel. Subsequently, the images of the blood vessels were analyzed and organized according to the genotypes of the larvae. For gene expression studies, sorafenib treatment of the *vhl^−/−^* mutants was conducted from 5 to 7 dpf. This timing was essential because a large number of *vhl^−/−^* larvae were required, and differentiating genotypes at earlier stages was challenging. So, the *vhl^−/−^* mutants were separated and sorafenib treatment was undertaken from 5 to 7 dpf. After 2 days of sorafenib treatment, the larvae were grouped into four categories for gene expression analysis: control, 0.2 μM sorafenib-treated group, *vhl^−/−^* mutant group, and the combined 0.2 μM sorafenib+*vhl^−/−^* mutant group.

### Statistics

Statistical analyses were performed using GraphPad Prism version 9 for Windows. The statistical tests applied included the unpaired *t*-test, one-way ANOVA with Tukey's multiple comparisons.

### Ethics declarations

Ethical approval was obtained from the Institutional Animal Ethics Committee (IAEC) of SRM institute of science and Technology, approval number: 16099/835re-S-04/IAEC 2016.

### Usage of AI assistance

We hereby declare that the content of this paper was written by the authors. ChatGPT was used solely to improve the readability and to verify the grammatical content.

## Supplementary Material

10.1242/biolopen.061863_sup1 Supplementary information
