# Supplementary material for: A pro-angiogenic and hypoxic zebrafish model as a novel platform for anti-angiogenic drug testing
Source: Biol Open. 2025 Aug 11;14(8):bio061863. doi: 10.1242/bio.061863 (PMC12381926; doi:10.1242/bio.061863)
Supplement: Supplementary information [file biolopen-14-061863-s1.pdf]

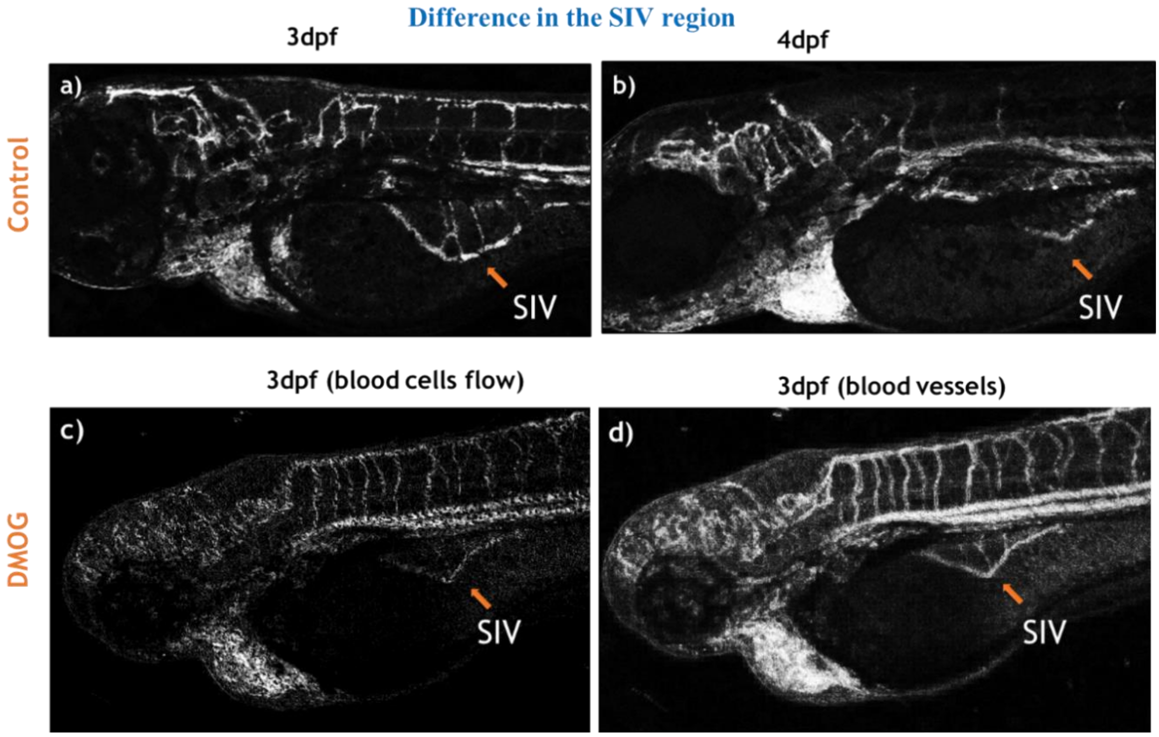


**Fig. S1.** Observation of blood vessel at (a) 3 and (b) 4 dpf zebrafish larva. (c) Blood cell flow and (d) blood vessels in DMOG treated 3 dpf zebrafish larva.

Biology Open • Supplementary information

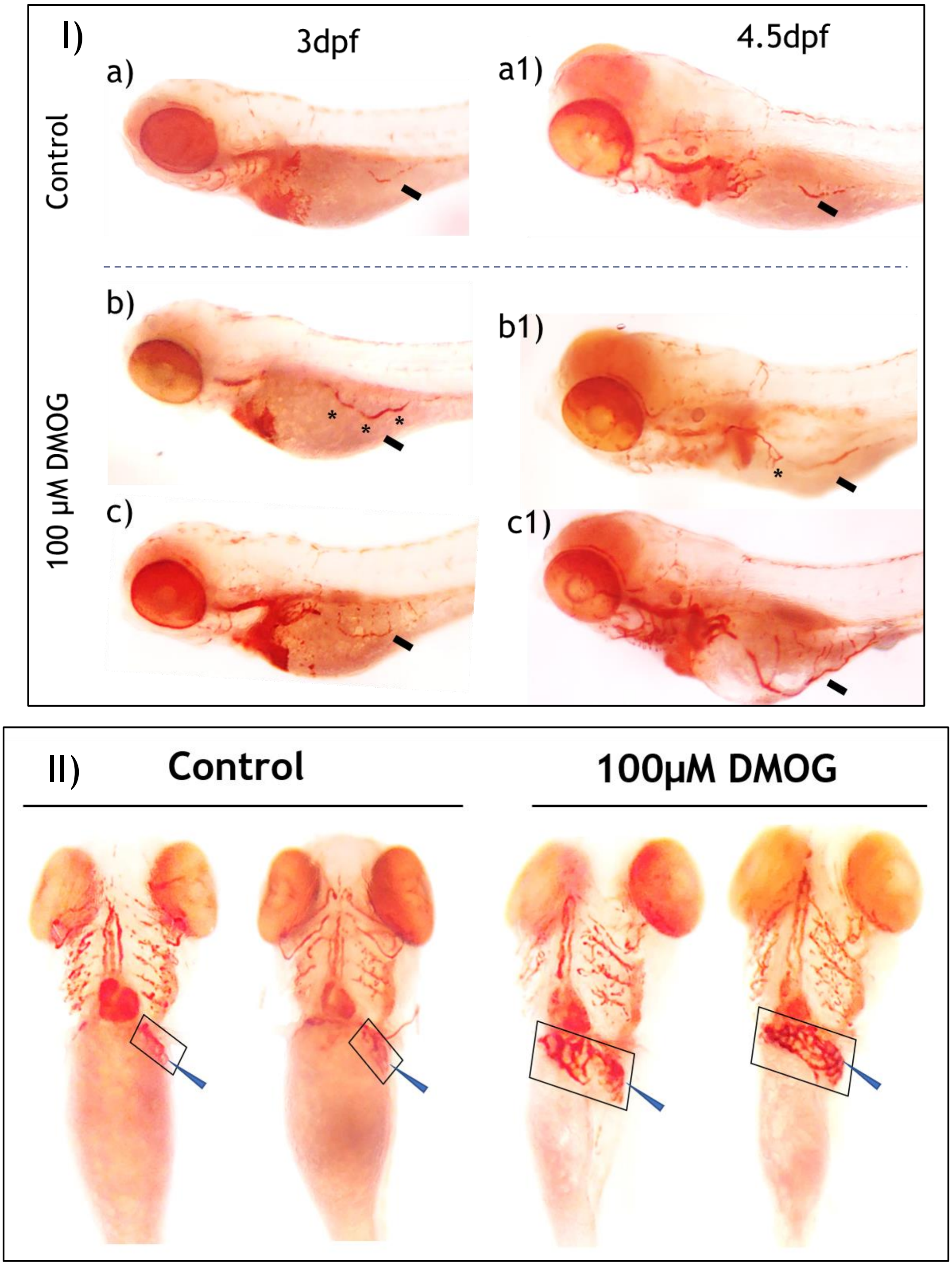


**Fig. S2.** Zebrafish larvae treated with DMOG (100µM) and (I) blood vessels were observed at 3 dpf: a and a1-control, b, c, b1, c1- are DMOG treated. (II) Zebrafish larvae treated with DMOG (100µM) showed excessive angiogenesis in liver region of 4 dpf zebrafish larvae. Astrix indicates the angiogenic sprouting. Images were taken in Olympus simple light microscope using mobile phone camera at 4X magnification.

Biology Open • Supplementary information

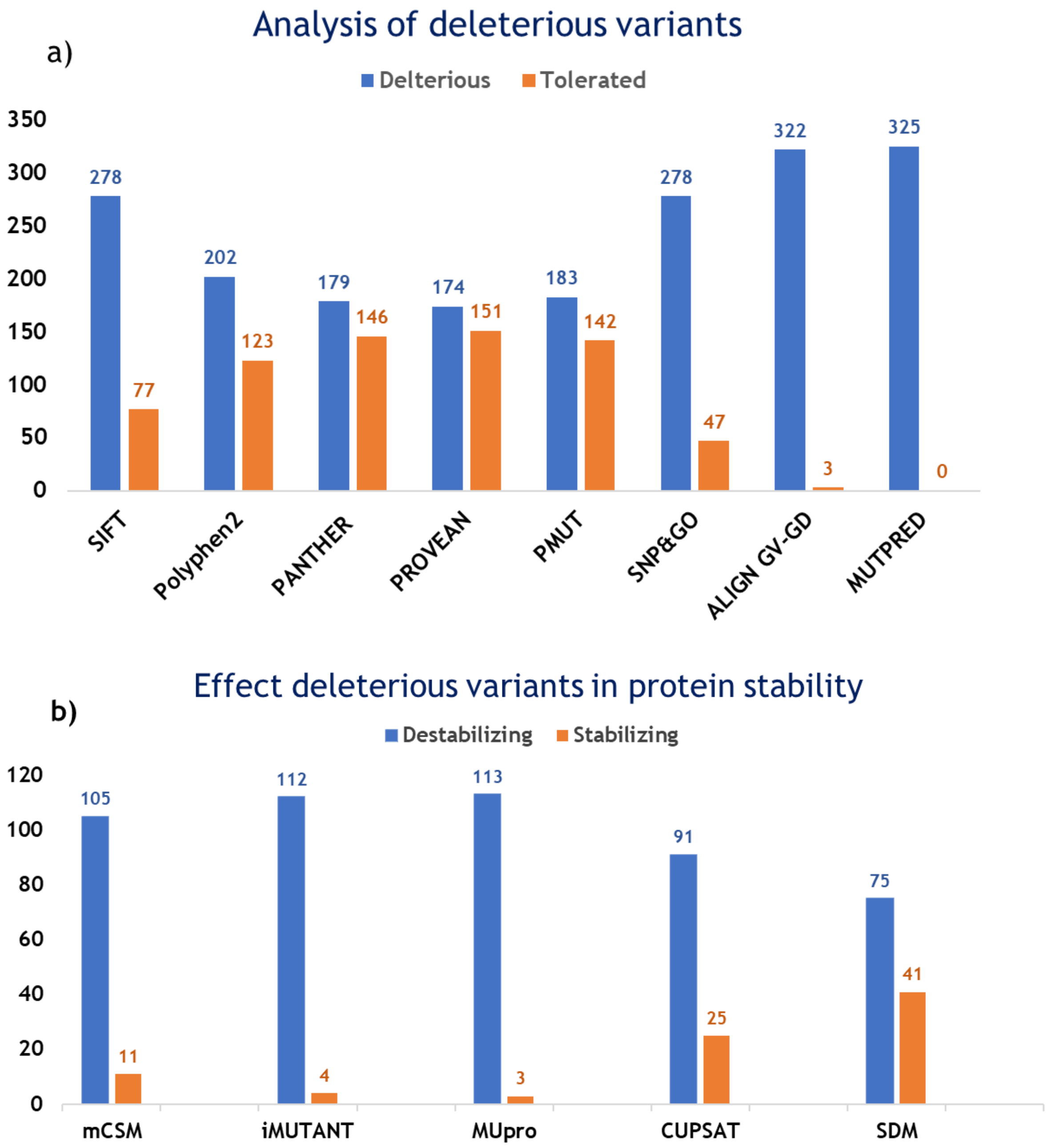


**Fig. S3.** (a) Screening and evaluation of deleterious SNPs in human *VHL* gene. (b) Analysis of effects of deleterious SNPs on VHL protein stability.

Biology Open • Supplementary information

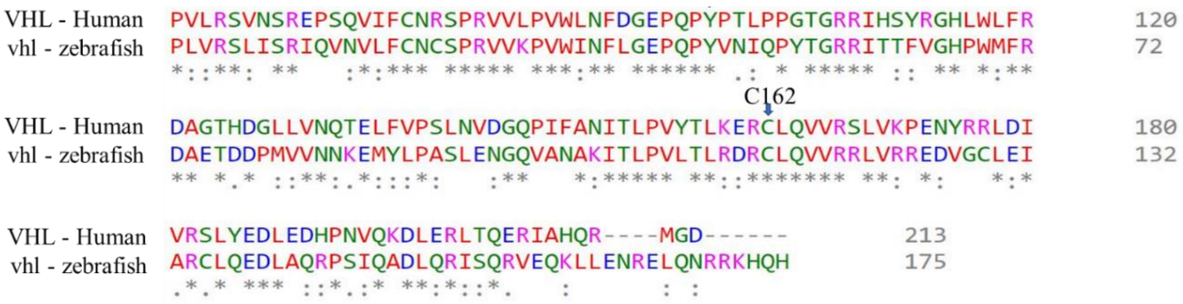


**Fig. S4.** Multiple sequence alignment of human VHL and zebrafish vhl protein sequence.

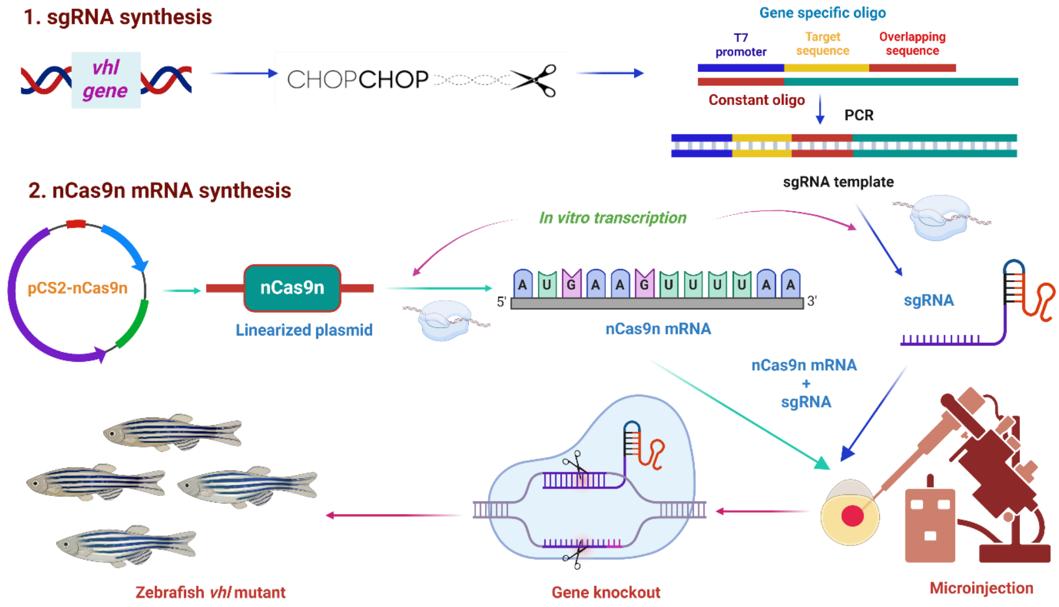


**Fig. S5.** Steps in generation of *vhl* mutant line using CRISPR mutagenesis. Created in BioRender by Chakraborty, P., 20205. https://BioRender.com/y5ydsy9. This figure was sublicensed under CC-BY 4.0 terms.

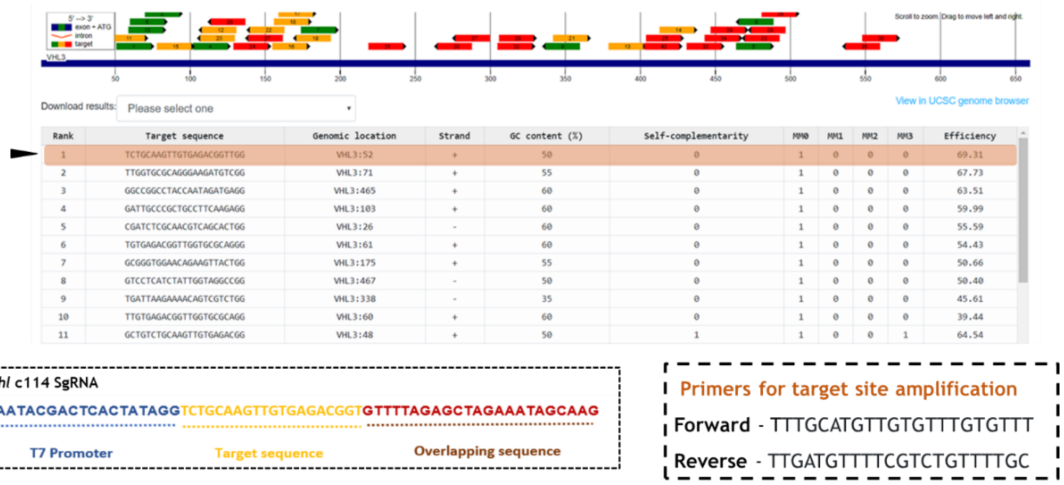


| Rank | Target sequence | Genomic location | Strand | GC content (%) | Self-complementarity | MM0 | MM1 | MM2 | MM3 | Efficiency |
|---|---|---|---|---|---|---|---|---|---|---|
| 1 | TCTGCAAGTTGTGAGACGGTTGG | VHL3:52 | + | 50 | 0 | 1 | 0 | 0 | 0 | 69.31 |
| 2 | TTGGTGCGCAGGGAAGATGTCGG | VHL3:71 | + | 55 | 0 | 1 | 0 | 0 | 0 | 67.73 |
| 3 | GGCCGGCCTACCAATAGATGAGG | VHL3:465 | + | 60 | 0 | 1 | 0 | 0 | 0 | 63.51 |
| 4 | GATTGCCCGCTGCCTTCAAGAGG | VHL3:103 | + | 60 | 0 | 1 | 0 | 0 | 0 | 59.99 |
| 5 | CGATCTCGCAACGTCAGCACTGG | VHL3:26 | - | 60 | 0 | 1 | 0 | 0 | 0 | 55.59 |
| 6 | TGTGAGACGGTTGGTGCGCAGGG | VHL3:61 | + | 60 | 0 | 1 | 0 | 0 | 0 | 54.43 |
| 7 | GCGGGTGGAACAGAAGTTACTGG | VHL3:175 | + | 55 | 0 | 1 | 0 | 0 | 0 | 50.66 |
| 8 | GTCCTCATCTATTGGTAGGCCGG | VHL3:467 | - | 50 | 0 | 1 | 0 | 0 | 0 | 50.40 |
| 9 | TGATTAAGAAAACAGTCGTCTGG | VHL3:338 | - | 35 | 0 | 1 | 0 | 0 | 0 | 45.61 |
| 10 | TTGTGAGACGGTTGGTGCGCAGG | VHL3:60 | + | 60 | 0 | 1 | 0 | 0 | 0 | 39.44 |
| 11 | GCTGTCTGCAAGTTGTGAGACGG | VHL3:48 | + | 50 | 1 | 1 | 0 | 0 | 1 | 64.54 |

**Fig. S6.** Designed templates for target sites of human *VHL* mutation C162 primers for target site amplification.

Biology Open • Supplementary information

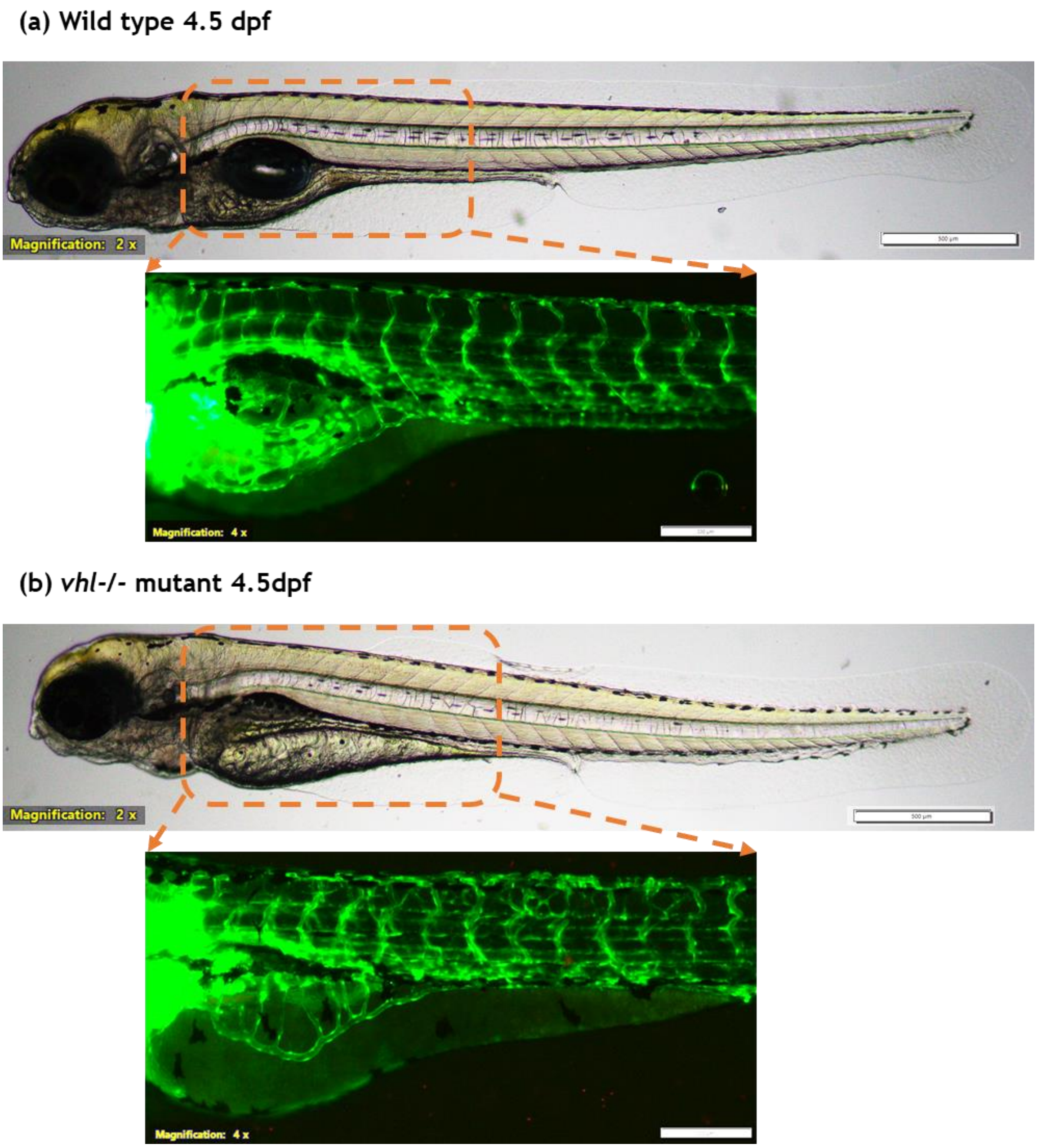


**Fig. S7.** Comparison of blood vessel at ISV of (a) wild type, (b) *vhl*$^{-/-}$ mutant at 4.5 dpf Tg(*fli1a*:*EGFP)* zebrafish larvae.

**Table S1.** Details of primer used in this study

| **S. NO** | **GENE** | **FORWARD** | **REVERSE** |
|---|---|---|---|
| 1. | *phd3* | ccggcttctgcttttctggtcag | aattcctctccgagacgggcttg |
| 2. | *actb1*-beta actin | ctgtacgcttctggtcgtactactg | ctcggtcaggatcttcatcaggtag |
| 3. | *gapdh* | gtggggtgatgcaggtgctact | ctgcacttggggcagagatgatg |
| 4. | *vegfab* | cgactgcaggtctaggcaactag | ctcccacatccatctccaaccac |
| 5. | *vegfc* | agagctggatgaggagacgtgtc | caggtgtccctgttgaagctctg |
| 6. | *vegfd* | gcaggtcaactcgatatgcagcc | cacctgaagacagagacgcatgg |
| 7. | *vegfaa* | cgagagctgctggtagacatcatc | gcacctccatagtgacgtttcgtg |
| 8. | *kdr* | gaccgcgtgtacacaacacagag | cgtggcgtaatctggagctctc |
| 9. | *kdrl* | ggtggagaggctaggagatttgc | gtcccagagtctctgagagagac |
| 10. | *flt1* | cgtcgttcaggcttttgcaggac | gcatcctcttctgcaacatcccg |

Biology Open • Supplementary information

**Table S2.** Ranking of mutations based on RMSD score. The wild type and the mutant column of the table represents single letter amino acid code.

| S. NO | WILD TYPE | MUTANT TYPE | POSITION | RMSD SCORE (Å) | RANK |
|---|---|---|---|---|---|
| 1. | C | W | 162 | 0.209 | 1 |
| 2. | C | F | 162 | 0.199 | 2 |
| 3. | S | P | 72 | 0.19 | 3 |
| 4. | S | R | 111 | 0.172 | 4 |
| 5. | L | P | 163 | 0.172 | 4 |
| 6. | L | Q | 178 | 0.17 | 5 |
| 7. | L | Q | 158 | 0.17 | 5 |
| 8. | L | P | 198 | 0.17 | 5 |
| 9. | I | S | 151 | 0.168 | 6 |
| 10. | P | A | 192 | 0.165 | 7 |
| 11. | Y | S | 98 | 0.165 | 7 |
| 12. | L | P | 64 | 0.165 | 7 |
| 13. | P | S | 192 | 0.164 | 8 |
| 14. | F | S | 76 | 0.164 | 8 |
| 15. | Y | H | 98 | 0.164 | 8 |
| 16. | H | R | 115 | 0.164 | 8 |
| 17. | D | N | 126 | 0.164 | 8 |
| 18. | L | R | 163 | 0.164 | 8 |
| 19. | Y | C | 98 | 0.163 | 9 |
| 20. | L | P | 169 | 0.161 | 10 |
| 21. | L | Q | 153 | 0.161 | 10 |
| 22. | V | A | 74 | 0.16 | 11 |
| 23. | I | T | 151 | 0.16 | 11 |
| 24. | G | C | 93 | 0.159 | 12 |
| 25. | L | R | 118 | 0.158 | 13 |
| 26. | P | A | 146 | 0.157 | 14 |
| 27. | L | P | 184 | 0.157 | 14 |
| 28. | F | C | 136 | 0.156 | 15 |
| 29. | R | C | 82 | 0.156 | 15 |

Biology Open • Supplementary information

| | | | | | |
|---|---|---|---|---|---|
| 30. | E | V | 160 | 0.155 | 16 |
| 31. | N | T | 78 | 0.155 | 16 |
| 32. | L | P | 118 | 0.154 | 17 |
| 33. | L | F | 118 | 0.153 | 18 |
| 34. | G | S | 93 | 0.153 | 18 |
| 35. | V | A | 130 | 0.152 | 19 |
| 36. | D | Y | 126 | 0.151 | 20 |
| 37. | P | R | 192 | 0.15 | 0.15 |
| 38. | Y | N | 185 | 0.15 | 0.15 |
| 39. | F | L | 136 | 0.149 | 0.149 |
| 40. | G | R | 144 | 0.149 | 0.149 |
| 41. | N | S | 78 | 0.148 | 0.148 |
| 42. | R | C | 79 | 0.148 | 0.148 |
| 43. | G | R | 93 | 0.148 | 0.148 |
| 44. | W | S | 88 | 0.147 | 0.147 |
| 45. | Y | N | 112 | 0.146 | 0.146 |
| 46. | Y | S | 185 | 0.144 | 0.144 |
| 47. | R | T | 182 | 0.144 | 0.144 |
| 48. | V | G | 74 | 0.144 | 0.144 |
| 49. | R | G | 120 | 0.143 | 0.143 |
| 50. | F | S | 136 | 0.143 | 0.143 |
| 51. | R | G | 167 | 0.141 | 0.141 |
| 52. | R | G | 182 | 0.14 | 0.14 |
| 53. | R | G | 161 | 0.14 | 0.14 |
| 54. | W | C | 117 | 0.14 | 0.14 |
| 55. | W | C | 88 | 0.14 | 0.14 |

Biology Open • Supplementary information

**Table S3.** Mutation in the β -domain of VHL and their conservation in zebrafish vhl protein. *Residues of β -domain interacts HIF were marked in red color.

| MUTATIONS IN B DOMAIN OF PVHL | RANKING BASED ON RMSD SCORE | CONSERVATION | NUMBER OF REPORTS | MUTATIONS IN B DOMAIN OF PVHL | RANKING BASED ON RMSD SCORE | CONSERVATION | NUMBER OF REPORTS |
|---|---|---|---|---|---|---|---|
| R64P | 7 | Yes | 6 | Y112N | 27 | Partial | 14 |
| S72P | 3 | No | 6 | H115R | 8 | Yes | 4 |
| V74A | 11 | Yes | - | W117C | 31 | Yes | 19 |
| V74G | 28 | Yes | 5 | L118F | 18 | Partial | - |
| F76S | 8 | Yes | 3 | L118P | 17 | Partial | 29 |
| N78T | 16 | Yes | - | L118R | 13 | Partial | 4 |
| N78S | 25 | Yes | 48 | R120G | 29 | Yes | 2 |
| R79C | 25 | No | - | D126N | 8 | Yes | 2 |
| R82C | 15 | Yes | 1 | D126Y | 20 | Yes | 4 |
| W88S | 26 | Yes | 9 | V130A | 19 | Yes | - |
| W88C | 31 | Yes | 4 | F136S | 29 | Partial | 17 |
| G93S | 18 | Yes | 6 | F136C | 15 | Partial | 7 |
| G93R | 25 | Yes | 4 | F136L | 24 | Partial | 1 |
| G93C | 12 | Yes | 2 | G144R | 24 | Yes | 2 |
| Y98H | 8 | Yes | 65 | P146A | 14 | No | - |
| Y98S | 7 | Yes | 12 | I151T | 11 | Yes | 9 |
| Y98C | 9 | Yes | 6 | I151S | 6 | Yes | 11 |
| S111R | 4 | Partial | 12 | L153Q | 10 | Yes | 1 |

**Table S4.** Mutation in the α -domain of VHL and their conservation in zebrafish vhl protein.
*Residues of α-domain interacts with elongin C were marked in red color

| MUTATIONS IN B DOMAIN OF PVHL | RANKING BASED ON RMSD SCORE | CONSERVATION | NUMBER OF REPORTS | MUTATIONS IN B DOMAIN OF PVHL | RANKING BASED ON RMSD SCORE | CONSERVATION | NUMBER OF REPORTS |
|---|---|---|---|---|---|---|---|
| L158Q | 5 | Yes | 8 | L178Q | 5 | Yes | 6 |
| E160V | 16 | Partial | 3 | R182G | 31 | Yes | Nil |
| R161G | 31 | Yes | 6 | R182T | 28 | Yes | Nil |
| C162F | 2 | Yes | 9 | L184P | 14 | Yes | 11 |
| C162W | 1 | Yes | 19 | Y185N | 23 | No | Nil |
| L163P | 4 | Yes | 5 | Y185S | 28 | No | Nil |
| L163R | 8 | Yes | 1 | P192A | 7 | Yes | - |
| R167G | 30 | Yes | 150 | P192S | 8 | Yes | 2 |
| L169P | 10 | Yes | 12 | P192R | 22 | Yes | - |

Biology Open • Supplementary information

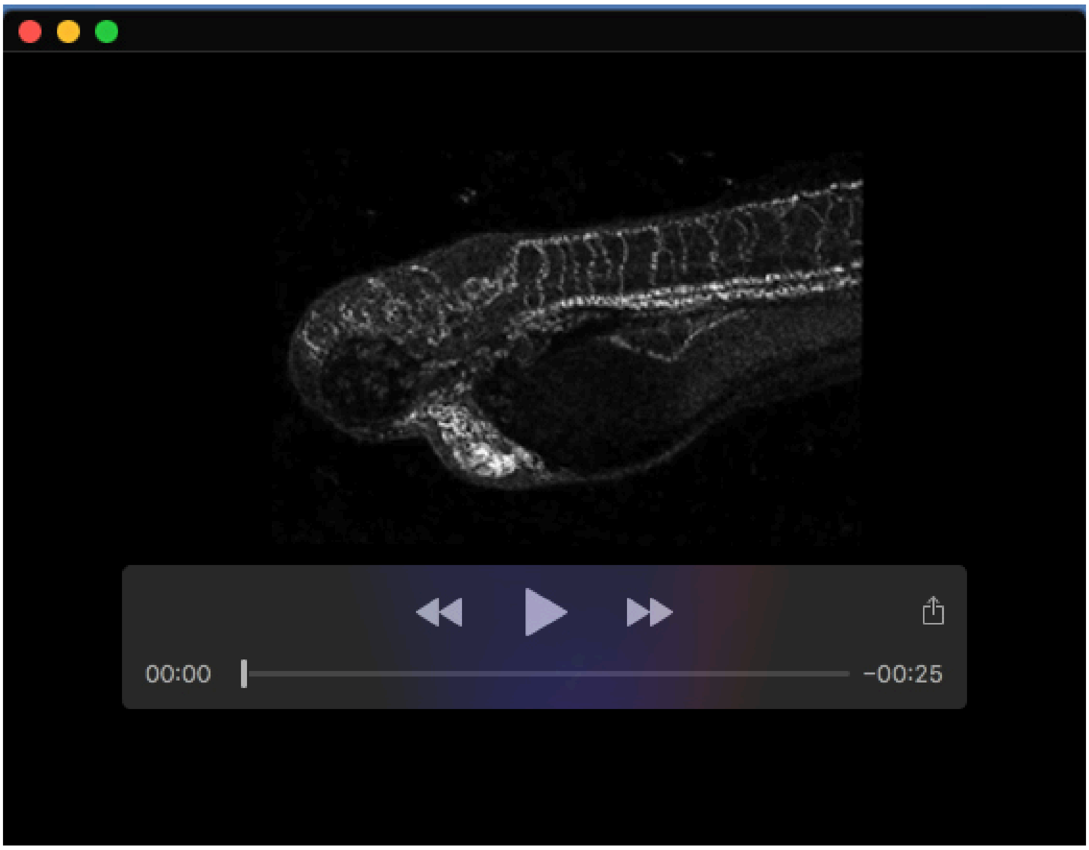


**Movie 1.**

Biology Open • Supplementary information
